# Improved LC–MS identification of short homologous peptides using sequence-specific retention time predictors

**DOI:** 10.1007/s00216-023-04670-2

**Published:** 2023-03-31

**Authors:** Boudewijn Hollebrands, Jos A. Hageman, Jasper W. van de Sande, Bauke Albada, Hans-Gerd Janssen

**Affiliations:** 1 Unilever Foods Innovation Centre – Hive, Bronland 14, 6708 WH Wageningen, the Netherlands; 2 Laboratory of Organic Chemistry, Wageningen University & Research, Stippeneng 4, 6708 WE Wageningen, the Netherlands; 3 Wageningen University & Research, Biometris, P.O. Box 16, 6700 AA Wageningen, the Netherlands

**Keywords:** Peptides, Identification, Retention time, Prediction, Sequence-specific descriptors

## Abstract

Peptides are an important group of compounds contributing to the desired, as well as the undesired taste of a food product. Their taste impressions can include aspects of sweetness, bitterness, savoury, umami and many other impressions depending on the amino acids present as well as their sequence. Identification of short peptides in foods is challenging. We developed a method to assign identities to short peptides including homologous structures, i.e. peptides containing the same amino acids with a different sequence order, by accurate prediction of the retention times during reversed phase separation. To train the method, a large set of well-defined short peptides with systematic variations in the amino acid sequence was prepared by a novel synthesis strategy called ‘swapped-sequence synthesis’. Additionally, several proteins were enzymatically digested to yield short peptides. Experimental retention times were determined after reversed phase separation and peptide MS^2^ data was acquired using a high-resolution mass spectrometer operated in data-dependent acquisition mode (DDA). A support vector regression model was trained using a combination of existing sequence-independent peptide descriptors and a newly derived set of selected amino acid index derived sequence-specific peptide (ASP) descriptors. The model was trained and validated using the experimental retention times of the 713 small food-relevant peptides prepared. Whilst selecting the most useful ASP descriptors for our model, special attention was given to predict the retention time differences between homologous peptide structures. Inclusion of ASP descriptors greatly improved the ability to accurately predict retention times, including retention time differences between 157 homologous peptide pairs. The final prediction model had a goodness-of-fit (*Q*^2^) of 0.94; moreover for 93% of the short peptides, the elution order was correctly predicted.

**Graphical abstract:**

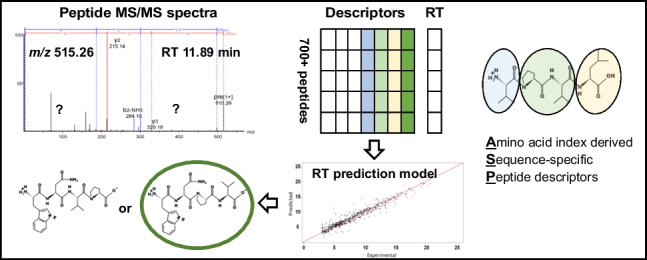

**Supplementary Information:**

The online version contains supplementary material available at 10.1007/s00216-023-04670-2.

## Introduction

Fermentation and hydrolysis are two commonly applied food preparation methods. From ancient times, they have been applied to preserve the food, to improve taste and structure or to make nutrients more available for bio-absorption. Well-known foods prepared using hydrolysis and fermentation are soy sauces or products such as cheese and yoghurt. Breakdown of the food proteins upon these treatments results in complex mixtures of peptides having different chain lengths and physico-chemical properties. Identification and quantification of these peptides is important to understand the flavour and taste characteristics of the products as well as to control its nutritional features. Peptides may taste bitter, umami, sweet, sour, salty or kokumi depending on the amino acid composition, their sequence and the higher-order peptide structure [1, 2]. Hence, they play a role in, e.g. the development of taste-full salt- or sugar-reduced healthier products. Oppositely, bitter peptides can potentially be an obstruction in the acceptance of plant-based meat and dairy alternatives. Clearly, information on the identity and levels of the smaller peptides present in foods is crucial.

The key technique nowadays in peptide identification is LC-MS. Although the characteristic peptide fragments in the MS/MS spectra in principle allow de novo sequencing of unknown peptides, modern LC–MS/MS methods for peptide identification often apply database searching for confident peptide and protein identification [3]. The latter process is especially successful when known proteins are hydrolysed with specific enzymes yielding larger peptides with specific, known end groups. Many studies have demonstrated successful application of LC-MS/MS analysis for peptide identification in foods. However, issues are still encountered in the identification of the smaller peptides, i.e. peptides with less than five amino acids [4]. Two main reasons can be identified; First, during MS/MS data-acquisition of these short peptides, poor quality spectra are often obtained because of over- or under fragmentation resulting in a lack of structural information and hence difficulties in database matching. Second, the limited structural diversity of short peptides results in a high likeliness of detecting isobaric ions in the same sample. As a result, spectral searches against protein databases for identification often return low confidence hits for short peptides.

Of all possible identification strategies to improve peptide identification [5], combining MS or MS/MS spectral information with retention time information is an attractive approach. Unfortunately, database storage of experimentally determined retention times is not really useful due to the massive number of potential peptides and the poor transferability of retention information between laboratories. Retention time prediction models have been proposed to solve this issue. Numerous models that predict the retention times of peptides have been published. These can be divided into three groups.

The classical methods either use a selection of peptide properties to derive quantitative structure-retention relationships (QSRR) [6, 7] or apply experimentally determined retention coefficients (RC) for each amino acid in the peptide sequence [8–12]. Although QSRR models and those based on RC of the individual amino acids in the peptide have shown a good performance in many applications, they have not been applied to the short peptides with taste-related properties which the food industry is interested in [1]. For these peptides, the high accuracy required for retention time predictions to aid peptide identification cannot be met, especially not for (isobaric) homologous peptide structures.

Several models have been developed that utilize sequence information as input (e.g. the amino acids positioned at C-term/N-term), next to the information of the amino acid composition. These models have mainly been applied to predict retention times for strong cation exchange [13] and hydrophilic interaction liquid chromatography separation [14]. An exception are the models developed by Krokhin et al. [8] which are intended for reversed phase chromatography. However, in that work trifluoroacetic acid (TFA) is added as an ion-pairing agent in the solvent system. TFA strongly stabilizes the retention of the more polar peptides resulting in improved prediction models. Unfortunately, the use of this additive is generally avoided in LC-MS analyses since it induces severe ion suppression [15]. When replacing TFA with formic acid, a more suitable additive for LC-MS analysis, the prediction models are no longer applicable [9].

Recently deep learning methods are applied for accurate retention prediction [16–18]. In these methods previously measured retention data is interpreted by multiple self-ensembled interpretation layers that also take the peptide sequence into consideration. Several of such models have been trained for retention time predictions of tryptic peptides of seven or more amino acids in length. Re-training these models requires large sets of data which currently do not exist for short peptides. Consequently, none of the strategies outlined above is suitable for prediction of the retention times of short homologous peptides structures.

In this paper, we developed a novel method for modelling retention times of short peptides in reversed phase LC separations. To achieve this, a large set of well-defined short peptides with systematic variations in the amino acid sequence was prepared by a novel synthesis approach called ‘swapped-sequence synthesis’. Moreover, a number of proteins were enzymatically digested to yield an additional range of short peptides. Experimental retention times of the peptides were determined after separation on a reversed phase LC column using formic acid as solvent additive and high-resolution accurate mass spectrometry as detection method. The retention times were used in combination with a set of existing ‘overall’ peptide descriptors as well as a set of newly derived amino acid sequence-specific peptide descriptors (ASP) to train a support vector regression model which utilized the key compositional and sequential information of the peptides. Whilst selecting the most useful ASP descriptors for our model, special attention was given to the accuracies in the prediction of the retention time differences between homologous peptide structures. Validation of the model was performed using an external peptide set. The model can be applied for improving the prediction of the retention times of short peptides, including homologous structures, in that way contributing to a more reliable identification of taste-relevant peptides in foods.

## Materials and methods

### Materials

Acetonitrile, methanol, formic acid and trifluoroacetic acid (ULC/MS grade) were obtained from Biosolve (Valkenswaard, the Netherlands). Dichloromethane and di-isopropylethylamine were purchased from Fisher Bioreagents (Basel, Switzerland). The amino acids used for peptide synthesis were purchased from Novabiochem (Nottingham, UK).

### Swapped-sequence synthesis

During synthesis, we focused on the amino acids that are commonly present in plant-proteins and that are considered most relevant in terms of taste and off-taste of plant-protein derived products. Amino acids known to be taste relevant, but not abundantly present in plant proteins such as cysteine (C), histidine (H), threonine (T) and methionine (M) were excluded. In total, five sets of peptide mixtures were prepared according to a published solid phase peptide synthesis protocol [19].

A tripeptide mix with 75 unique peptides was synthesized in a three-step operation. One gram of 2-chlorotrityl chloride resin (Iris Biotech GmbH, Marktredwitz, Germany) was used (loading capacity 0.8 mmol). Resin was preswollen with dichloromethane (DCM) and subsequently treated with 0.3 Eq. (0.24 mmol) of each Fmoc-Arg(Pbf)-OH (R), Fmoc-Pro-OH (P) and Fmoc-Phe-OH (F) in DCM with 2 Eq. (1.6 mmol) di-isopropylethylamine (DIPEA) for 2 h. Non-reacted trityl groups were capped using methanol (wash 3 × 15 min with 17:2:1 DCM:MeOH:DIPEA). Afterwards, the beads were washed with DCM (3 × 2 min) and DMF (2 × 2 min) and dried using diethyl ether. Fmoc was removed using 20% piperidine in N,N-dimethylformamide DMF (2 × 8 min). Then the resin was washed with DMF (3 × 2 min). After this, Fmoc-Gly-OH (G), Fmoc-Ala-OH (A), Fmoc-Lys(Boc)-OH (K), Fmoc-Trp(Boc)-OH (W) and Fmoc-Leu-OH (0.2 eq each, 0.16 mmol) were activated with 2-(1H-benzotriazole-1-yl)-1,1,3,3-tetramethyluronium hexafluorophosphate (HBTU) (1 eq, 0.8 mmol) and DIPEA (2 eq, 1.6 mmol) in DMF for 2 min before being added to the resin. The reaction mixture was allowed to couple for 2 h. Successful acylation was proven by resin staining using ninhydrin (15 g/L, supplemented with 30 mL/L acetic acid in n-butanol). Upon completion, the resin was washed with DMF (3 × 2 min) and deprotected by 20% piperidine in DMF (2 × 8 min). After washing again with DMF (3 × 2 min), the next amino acids were coupled repeating the same process. For this coupling, Fmoc-Val-OH (V), G, K, L and A (0.2 eq each, 0.16 mmol) were used in order to obtain a mixture with 75 unique peptides.

Four sets of tetrapeptide mixtures were prepared with in total 216 unique tetrapeptides in all four sets combined. Similar to the approach of the tripeptide mixture, 1 g of 2-chlorotrityl chloride resin was used. The first three amino acids (0.3 eq each) were coupled in a similar way. For set 1: G, Fmoc-Ile-OH (I) and V were used; for set 2: Fmoc-Gln(Trt)-OH (Q), Fmoc-Asn(Trt)-OH (N) and Fmoc-Ser(tBu)-OH (S) were used; for set 3: W, Fmoc-Tyr(tBu)-OH and A were used; for set 4: Fmoc-Glu(OtBu)-OH (E), Fmoc-Asp(OtBu)-OH (D) and Fmoc-Thr(tBu)-OH (T) were used and reacted for 2 h. Non-reacted trityl groups were capped, also with methanol mixture, and subsequently washed. The four mixtures were then reacted with the C1 amino acids, for sets 1–2: P, T and K, and for sets 3–4: R, I and G (0.333 eq of each amino acids, 0.27 mmol). For this coupling, HBTU (1 eq, 0.8 mmol) and DIPEA (2 eq, 1.6 mmol) in DMF were used. The same procedure was repeated for the coupling on the C3 position: for set 1: Q, N, S; for set 2: G, I, V; for set 3: E, D, T; and for set 4: W, Y, A. The fourth and final N-terminal coupling included coupling of 2 amino acids per mixture: for sets 1–2: W, Y, and for sets 3–4: P, F. 0.5 eq of each peptide was used (0.4 mmol), and again 1 eq of HBTU and 2 eq of DIPEA in DMF. Each peptide coupling was reacted for 2 h.

The formed peptides were acidolytic cleaved from the resin and fully deprotected by treatment with a cocktail of 95% trifluoroacetic acid (TFA), 2.5% triisopropylsilane (TIS) and 2.5% Milli-Q (deionized water, produced with a Milli-Q Integral 3 system; Millipore, Amsterdam, the Netherlands) in a ratio of 10 mL/1 g resin for 3 h. Each cleaved peptide resin was then washed extensively with fresh cleavage cocktail. The peptide was precipitated by addition of ice cold diethyl ether (1:1 ether:hexane, 10 × initial cocktail volume) and centrifuged for 10 min at 6000 rpm. The supernatant was discarded, and the precipitate was washed with ice-cold diethyl ether and again centrifuged for 10 min at 6000 rpm. The wash step was repeated once more. The resulting precipitate was dried under a light stream of N2, redissolved in acetonitrile (ACN):Milli-Q (4:6) and then lyophilized (Labconco FreeZone lyophilizer, 2.5 L,  − 84 °C, connected to a 35i xDS Edwards Oil-Free Dry Scroll Pump). Since the standards were only used for qualitative analysis, the obtained crude peptide mixtures were used without any further purification, and no further purity assessments were performed.

In Table 1, the synthesis route of the homologous tetrapeptides is shown. For the generation of the peptides with altered amino acid sequences, the steps 1 and 3 in the synthesis procedure were swapped.

**Table 1:**
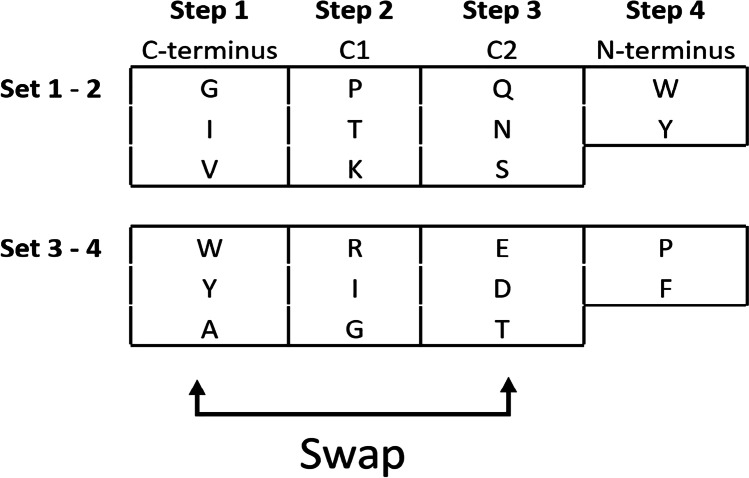
Scheme for the swapped-sequence synthesis

### Protein digestions

Albumin from chicken egg white, α-lactalbumin from bovine milk and κ-casein from bovine milk were obtained from Sigma (Zwijndrecht, the Netherlands). Papain enzyme from *Carica papaya* (10 mg/mL) and TFA was purchased from Merck (Hohenbrunn, Germany). The protein standard solutions were prepared by dissolving 1 mg of the respective protein in 1 mL demineralized water (Millipore, Amsterdam, the Netherlands) in individual Eppendorf tubes. Standard solutions were pre-incubated with 30 µL of papain solution (1 mg/mL) at 65 °C for 5 min. The enzyme-to-protein ratio was 1:33. The sample was incubated overnight (16 h) at 37 °C. Enzyme deactivation was then performed by heating the solutions to 95 °C for 5 min.

### Peptide standard solutions

Ten milligram of each of the five synthesized peptide mixes was accurately weighed into individual 15-mL Falcon tubes. The standards were dissolved in 1.0 mL solution of 10% acetonitrile, 1% trifluoroacetic acid and 89% Millipore water (v/v). The expected concentrations in the stock solution per peptide were approximately 0.185 g/L or 370 µM (10 mg/54 peptides per mL, assuming an average MW of 500 Da). The stock solutions were further diluted prior to analysis.

### Reversed-phase liquid chromatography–UV absorption–high-resolution mass spectrometry–ddMS^2^

All analyses were performed on an UltiMate 3000 RS chromatography system equipped with a UV detector, connected to a Q-Exactive Plus Hybrid Quadrupole-Orbitrap mass spectrometer (Thermo Fischer Scientific, Waltham, MA, USA). An Xselect Peptide CSH C18 130 Å column (particle size 2.5 µm, dimensions 150 × 2.1 mm, Waters, Etten-Leur, the Netherlands) with associated VanGuard pre-column was used for the separations. The analytes were eluted at a flow rate of 0.35 ml/min using a linear gradient of water (solvent A) and acetonitrile (solvent B) both fortified with 0.1% formic acid. The gradient was programmed as followed: 1 min at 1.0% B, in 29 min to 40% B, in 3 min to 100% B, 3 min at 100% B, in 1 min back to 1.0% B and finally re-equilibration for 11 min at 1.0% B. The total run time was 50 min. The column temperature was maintained at 40 °C and the auto-sampler at 4 °C. The injection volume was 5 µL. UV absorption was measured at wavelengths 214 and 280 nm with a bandwidth of 4 nm at a frequency of 5 Hz.

The heated electrospray ion source was operated in positive mode at a capillary temperature of 300 °C and a heater temperature of 413 °C. The sheath gas was set to 48 arbitrary units, the auxiliary gas was set to 11 arbitrary units, and the spray voltage was set to 3.7 kV. The mass spectrometer was set to operate in full-scan MS data-dependent MS^2^ (ddMS^2^) mode. Full-scan spectra were acquired at a resolution of 70.000 in the *m/z* range 80–1200 using a maximum ion injection time of 100 ms, unless stated otherwise. From the top five most abundant ions, ddMS^2^ scans were acquired at a resolution of 17.500 with an isolation window of 2 m/z whilst having the dynamic exclusion list set to 10 s. The maximum injection time was set to 150 ms, and a normalized stepped collision energy was applied of 15, 30 and 45 arbitrary units.

### Peptide identification

The peptides in the digest were identified by de novo peptide sequencing and database searching using Peaks 8 software (Bioinformatics Solutions Inc., Waterloo, Ontario, Canada) [20]. Search parameters were specified enzyme: none; precursor ion mass error tolerance: 10 ppm; fragment ion error tolerance: 0.5 Da; dynamic modifications: none; peptide multiple charges from 1 + to 3 + ; and monoisotopic precursor mass. To reduce the number of false positive identified peptides, the hit threshold (− 10logP) was set at  ≥ 15, and the de novo score (average local confidence) threshold was set to the value of 70.

The reported amino acid sequences of tentatively identified short peptides with low reported confidence scores were manually compared with the amino acid sequence of the digested protein standards. This included the manual validation of the MS/MS spectra and the proposed peptide sequences. Only small peptides that had a sequence that occurred in the protein were included in the final peptide dataset. This approach resulted in approximately 450 identified peptides from the digested proteins with a sequence length of 5 or less amino acids and their experimental retention times. A list of all identified peptides sequences is added in Table S1, in the supporting materials.

### Data processing

Peptide retention times were modelled using peptide descriptors as explanatory variables. The LC-MS dataset, Table S1, used for building the prediction models was randomly split in a training set and test set using an 85:15 ratio. All training data was centred and scaled to unit variance prior to training of the model, and a tenfold bootstrap cross-validation was applied. Support vector regression (SVR) [21] was used for creating the model and was implemented in R-studio (version 2021.09.0 build 351) using the SVMradial function from the R-package Caret (version 6.0–92) [22]. The method uses the following hyper parameters: cost-value and sigma. The hyperparameters were optimized by a grid search, and the final optimized model had a cost and sigma value of 14 and 0.01, respectively. The model file and a R-script to perform retention time predictions can be found at github.

### Descriptors

**Fig. 1:**
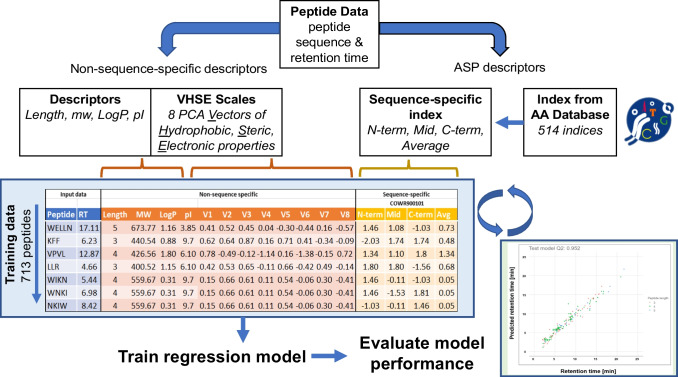
Schematic representation of the peptide descriptor information used to build and evaluate retention models for short peptides. For each peptide, a standard set of non-sequence-specific descriptors was calculated next to a set of sequence-specific descriptors utilizing the amino acid index database. All models were evaluated on their performance

Two types of peptide descriptors were used in the model as explanatory variables, first non-sequence-specific descriptors and secondly amino acid index derived sequence-specific peptide (ASP) descriptors, Fig. 1.

#### Non-sequence-specific peptide descriptors

The non-sequence-specific descriptors included in the model are the molecular weight, peptide length, the average calculated hydrophobicity according to the Abraham-Leo scale [23], the calculated isoelectric point using the emboss scale [24] and eight principal components score vectors of the hydrophobic, steric and electronic properties [25]*.* These descriptors were calculated for each peptide sequence using the R-package peptides (version 2.4.4) [26].

#### Amino acid index derived sequence-specific peptide (ASP) descriptors

In total, 514 amino acid indices reported in the AA index database [27] were evaluated as relevant descriptors (indices with missing values were excluded). Using each amino acid index listed in this database, four sequence-specific descriptors were calculated for each peptide sequence in the dataset. The first descriptor represents the amino acid positioned at the N-terminus, the second represents the amino acid at the C-terminus, the third value represents the average of the derived indices of the amino acid(s) between the N- and C-terminus, and the last value is the calculated average of all amino acids in the sequence.

### Step-wise descriptor selection

A prediction model was generated for each combination of non-sequence-specific descriptors and ASP descriptors. A goodness-of-fit value for the test set (*Q*^2^) [28] was calculated to evaluate if the added ASP descriptors had a positive contribution to the retention time predictability. Best performing ASP descriptors were added to the model in a step-wise manner. ASP descriptors were added to the model based on the improvement they gave to the model. A maximum of four ASP descriptors were added to the non-sequence descriptors.

### Evaluating model performance for homologous peptides

All prediction models were evaluated on their goodness-of-fit value (*Q*^2^) as well as on their ability to predict the retention time difference between homologous peptide structures synthesized in house using the swapped-sequence method. The experimental retention time differences (ΔRT_exp_) and predicted retention differences (ΔRT_pred_) between homologous pairs were calculated for all sets of homologous peptides present in our dataset. For each set, the retention time of the last eluting peptide was subtracted with the retention time of the first eluting peptide. Using the same elution order, the ΔRT_pred_ was calculated with the predicted retention times obtained from the model. The goodness-of-fit-value *Q*^2^_ΔRT_, which compares ΔRT_pred_ with ΔRT_exp_ for the homologous peptide structures, was calculated to evaluate the performance of the ASP descriptors for specifically homologous pairs.

## Results and discussion

**Fig. 2:**
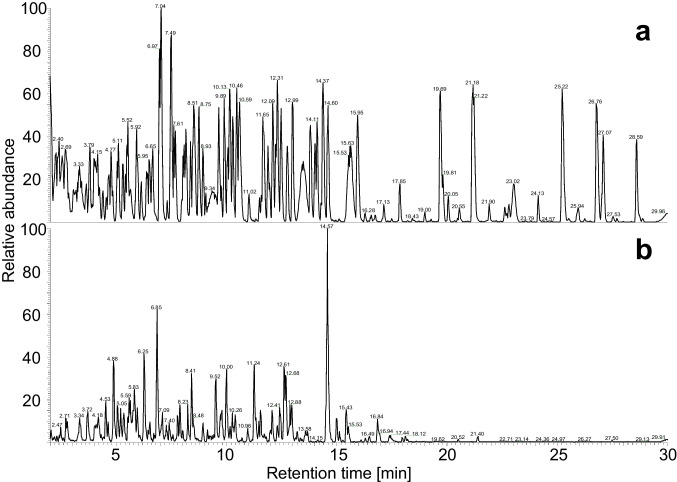
LC-MS base-peak ion chromatograms of a synthesized tetrapeptide mix obtained by swapped-sequence synthesis (**a**) and papain digested albumin (**b**) recorded in positive ionization mode. Mass range scanned 150–1200 m/z

Development of a retention prediction model requires a large set of retention data for training, preferably originating from peptides with identical amino acid compositions but with different sequences. As such peptides are difficult to get from natural sources, a sample set was generated using a novel synthesis strategy we call ‘swapped-sequence’ synthesis. Sets of tri- and tetrapeptides were synthesized using solid phase peptide synthesis with a focus on the generation of homologous peptide structures with swapped amino acid sequences. The combined synthesized peptide sets include multiple mixed samples with a theoretical total of 345 peptides, including 108 pairs of homologous tetrapeptides. The synthesized peptide standards were analysed using reversed phase LC high-resolution accurate mass MS to separate and identify the peptides. As an example, in Fig. 2a, the LC-MS profile is shown of one of the synthesized tetrapeptide mixes (calculated maximum number of tetrapeptides present 54).

For each synthesized peptide that we expected to be present in each standard mixture, the molecular weight was calculated, and the peptides were identified by searching the specific masses of the singly or doubly charged protonated molecular ions. In case identification was not straightforward, for example, when isobaric peptides were present in one of the measured standard mixtures, the peptide fragmentation spectra were used for identity assignment.

In total, 200 tripeptides and 188 tetrapeptides were found in all synthesized peptide mixes. This is slightly higher than the theoretical total number of peptides synthesized. Missed linkages during (tetra-)peptide synthesis resulted in the formation of additional tripeptides.

More short peptides were generated by enzymatic digestion of albumin, α-lactalbumin and κ-casein protein with papain. LC-MS analysis of these digests, an example is shown in Fig. 2b, resulted in an additional list of 147 identified short peptides. These included some auto-hydrolysis peptides from papain. The number of identified peptides in the papain digests is low in comparison to the number of peaks seen in the LC-MS chromatogram. The smaller peptides generally lack fragment ions in their MS/MS spectra, making both determination of the amino acid composition and their sequence impossible. These smaller peptides are very important from the taste perspective, so the data was also manually evaluated to increase the number of identified small peptides. This eventually resulted in approximately 450 peptides from the digested proteins with a sequence length of 5 or less amino acids and their experimental retention times. In Table S1, all peptides and their respective retention times are displayed.

The average retention time of the synthesized peptides was 7.6 min, which was just slightly lower than the average retention time of 7.8 min for the peptides originating from the protein digests. This indicates a similar overall hydrophobicity of the synthesized- and protein-digest peptides. Peptides that eluted very early from the LC column (< 3 min) have minimal interaction with the LC-column, and therefore their retention time is difficult to predict. Such peptides were excluded from the final set. The final peptide dataset from all sources combined contained 269 tripeptides, 360 tetra-peptides and 84 penta-peptides. The final list of peptide data used to build the model is given in the Supporting Material.

### Predictive retention time model for short peptides

A support vector regression model was trained based on 12 non-sequence-specific descriptors resulting in a model with a mean goodness-of-fit value (*Q*^2^) of 0.87 for the validation data with a standard error of 0.0035 out of 50 replicate runs. This performance is reasonable and compares well with performance values reported in similar studies for retention time prediction of large peptides [11, 12, 29]. Nevertheless, the experimental LC-MS data recorded in the current study clearly indicate that the retention time of homologous peptide structures can differ significantly in reversed phase chromatography. As an example, in our experimental data from the swapped-sequence peptides, the tri-peptide SPI has a retention time of 5.97 min, whereas its homologue IPS has a retention time of only 4.94 min. The retention time predicted using the method that did not take into account the amino acid sequence evidently is the same for both peptides. With our non-sequence specific model, it was 5.06 min. Similarly, the tetrapeptide YITS and its homologue YSTI have retention times of 6.84 and 8.34 min, respectively. With the non-sequence-specific model, the predicted retention time for these peptides is 8.30 min. The amino acid sequence clearly has a significant impact on retention in chromatography, and the results convincingly indicate that it is insufficient to use just the information on which amino acids are present in a peptide for prediction of the retention time [30]. Consequently, models that do not include sequence information cannot achieve the accuracy required for truly improving the reliability of peptide identification methods employing retention time prediction [14].

Our method calculates sequence-specific peptide descriptors derived from the amino acid index database, here coined ASP descriptors. The amino acid indices reported in the AA index database [27] were evaluated as a potential means to improve retention time prediction. For each AA index in the database, firstly, the ASP descriptors were calculated; secondly, the ASP descriptors were added to the non-sequence-specific descriptors; thirdly, SVM prediction models were generated (514 models in total); and finally performance of the retention time prediction model was evaluated using goodness-of-fit (*Q*^2^).

**Fig. 3:**
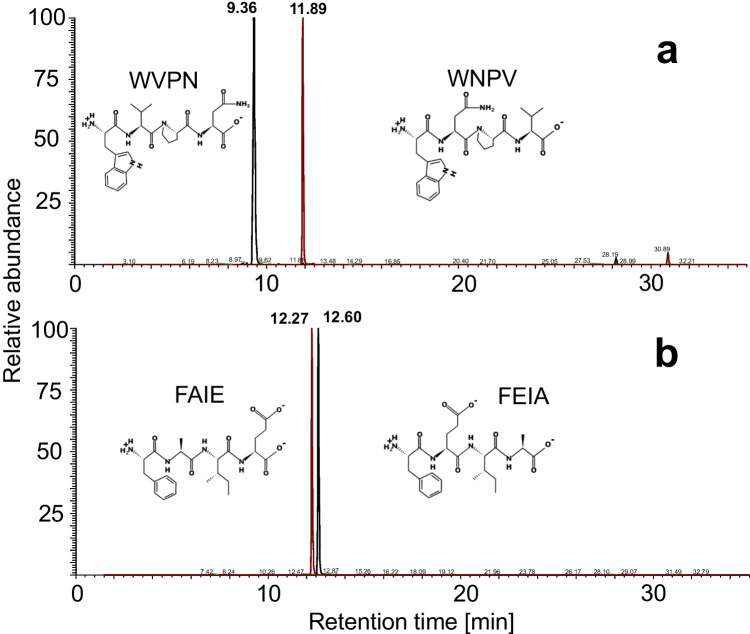
Experimental retention time differences between selected short homologous peptides. The extracted ion chromatograms of WVPN/WNPV 515.26 *m/z* (**a**) and FAIE/FEIA 479.25 *m/z* (**b**)

The *Q*^2^ values of the models with one added ASP descriptor ranged from 0.87 to 0.94, the latter being a major improvement in the ability to accurately predict retention times. For numerous models, little to no improvement was obtained in comparison to the performance of a model without ASP descriptors. In total, 279 models showed an improvement in performance by having *Q*^2^ values above 0.90 compared to 0.87 for the model without sequence information. This indicates that the predicted retention times are closer to the experimental values of the test data of the model. Whilst the goodness-of-fit value (*Q*^2^) is an informative performance indicator for models, limited information is obtained on the performance to predict retention times of homologous peptide structures. As outline before, such information is extremely important from a taste perspective. In our experiments, the retention time differences we observed between the homologous peptide pairs varied in magnitude and were sequence dependent. As an example, Fig. 3 shows two peptide pairs with large (3a) and small (3b) differences in the experimental retention times. The peptide WVPN had a retention time of 9.36 min; its homologue WNPV eluted at 11.89 min, resulting in an experimental retention time difference (ΔRT_exp_) of more than 2.5 min. For the homologous peptide pair FAIE and FEIA, only a small ΔRT_exp_ of 0.3 min was observed. Note that prediction models that do not include sequence information would have predicted the same retention time for both homologues.

The experimental retention time differences (ΔRT_exp_) were calculated for all 168 homologue peptide pairs in our dataset. Most of these homologous peptide pairs originated from the swapped-sequence peptide synthesis. The ΔRT_exp_ values were compared with the predicted retention time values (ΔRT_pred_) for all 514 models with ASP descriptors. The suitability of the model to predict these differences in the retention time between homologous peptides was expressed as the *Q*^2^_ΔRT_ score, where a score of close to 1 reveals perfect correlation between ΔRT_pred_ and ΔRT_exp_. The calculated baseline *Q*^2^_ΔRT_ score for the model without ASP descriptors was -1.66. This clearly indicates that this model is not capable to predict retention time differences between homologous peptides.

**Fig. 4:**
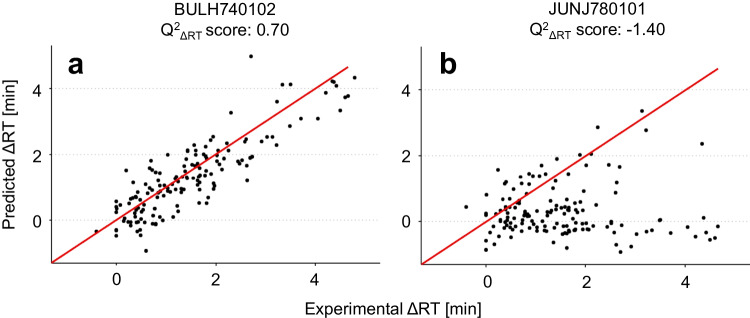
The predicted retention time differences (ΔRT_pred_) of 157 homologous tri- and tetrapeptide pairs plotted versus the experimental retention time differences (ΔRT_exp_) of a model with a *Q*^2^_ΔRT_ score of 0.70 (**a**) and *Q*^2^_ΔRT_ score of  − 1.40 (**b**)

Inclusion of ASP descriptors in our models showed in many cases an improvement in the prediction of ΔRT_pred_. In Fig. 4, the ΔRT_exp_ of homologous tri- and tetrapeptide pairs are plotted versus ΔRT_pred_ for two models using a good and bad ASP descriptor. A model that was trained on the ASP descriptors derived from the amino acid indices established by Bull-Breese [31] (entry BULH740102 in the database) showed a good agreement between ΔRT_exp_ and ΔRT_pred_: a calculated *Q*^2^_ΔRT_ score of 0.70 was obtained for this model (Fig. 4a). On the contrary, the model that uses the ASP descriptor based on the amino acid index derived from Jungck [32] (entry JUNJ780101 in the database) (Fig. 4b) shows no correlation between ΔRT_pred_ and ΔRT_exp_ and has a low *Q*^2^_ΔRT_ score of -1.40, which is a negligible improvement compared to the baseline.

For 65 models out of the 514 models generated, *Q*^2^_ΔRT_ scores above 0.55 were obtained if ASP descriptors were included. The strongest improvements to the *Q*^2^_ΔRT_ scores were obtained when ASP descriptors that were derived from one of the numerous hydrophobicity indices listed in the amino acid index database, such as BULH740102 [31] in Fig. 4a, were included in the models. These other hydrophobicity indices are reported in the work of Bastolla [33], Cowan [34], Ponnuswamy [35] and Manavalan [30]. In general, the elution order for homologous peptide structures was found to be strongly correlated to the hydrophobicity of the C-terminal amino acid. A similar finding was reported by Tripet et al. for separation of peptides in reversed phase chromatography using TFA as ion-pairing agent [36].

When adding additional ASP descriptors and generating a new set of models, 44 models were obtained with *Q*^2^_ΔRT_ scores above 0.73. In doing so, the use of stepwise variable selection ensures that only ASP descriptors complementary to each other are added to the model. Addition of a third set of ASP descriptors did not significantly improve model performance and inclusion of more than three ASP descriptors resulted in overfitting of the model and was therefore not further explored. The final model used a combination of the two ASP descriptors derived from the amino acid indices BULH740102 and QIAN880111, shown in Table 2. This model had *R*^2^, *Q*^2^ and *Q*^2^_ΔRT_ values of 0.97, 0.94 and 0.77 respectively.

**Table 2:** Two selected amino acid indices used to calculate the ASP descriptors

	A	R	N	D	C	Q	E	G	H	I
BULH740102	0.691	0.728	0.596	0.558	0.624	0.649	0.632	0.592	0.646	0.809
QIAN880111	0.21	0.07	− 0.04	− 0.58	− 0.12	0.13	− 0.23	− 0.15	0.37	0.31
	L	K	M	F	P	S	T	W	Y	V
BULH740102	0.842	0.767	0.709	0.756	0.730	0.594	0.655	0.743	0.743	0.777
QIAN880111	0.70	0.28	0.61	− 0.06	− 1.03	-0.28	-0.25	0.21	0.16	0.00

The model predicted the elution order correctly for 93% of all peptide pairs. Exceptions were only found for peptide pairs where the experimental retention time differences were less than 0.4 min. In daily use of the retention time prediction models, not only the predicted absolute retention time is useful, but especially the prediction of the elution order of different peptide sequences having the same amino acid composition is relevant for verification of a peptide structure. Since a main difficulty in the LC–MS identification of small peptides is establishing the amino acid order, accurate prediction of relative elution times or elution orders is even more important than a high accuracy in absolute retention times. Although the prediction of the elution order is not flawless, the model is a major improvement in comparison to any model without sequence specific information.

**Fig. 5:**
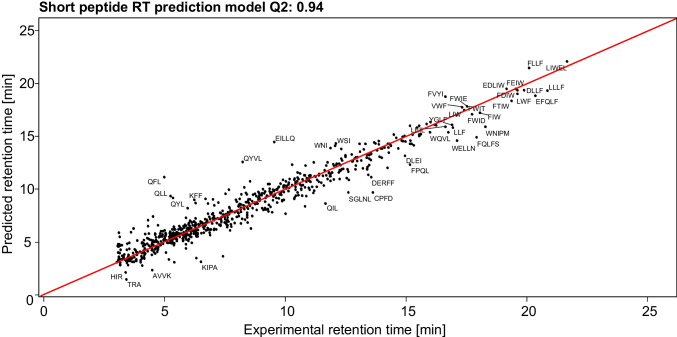
Experimental versus predicted retention time of our model

In Fig. 5, the retention times predicted with our model using the selected ASP descriptors are plotted versus the experimental retention times. For most peptides, there is an excellent correlation between the predictions and the experimental retention times. Although the artificial intelligence methods used to derive our models are to some extent black boxes, the models often were found to have a sound physico-chemical basis. During LC-separation at low pH, the N-terminal amino acid is charged; consequently, there is an increased likeliness that the C-terminal amino acid will determine the interaction with the hydrophobic column material [36]. This interaction seems especially important for short peptides. For peptides that contain an additional charged amino acid in their sequence, this simple retention rule seems no longer to apply, and it is more difficult to predict how the peptide will interact with the column material. As a result, retention times of the peptides with a charged group (K, R and Q) are more difficult to predict. Addition of indices into the model that specifically target this could potentially improve the predictability. Moreover, this also applies to leucine and isoleucine isomerism. However, due to the limited number of peptides in our sample set having charged groups, no relevant descriptors were found here. It is expected that the predictability of peptides with charged groups could be improved if more peptides with charged groups are included in the dataset.

Alternatively, the use of a strong ion-pairing agent could minimize the contribution of the charged amino acid residues on retention. An ion-pairing agent would also interact with the charged N-terminal amino acid which would result in a very different retention mechanism for all peptides, one that more depends on the average hydrophobicity of peptides after shielding all charged groups. The latter would make the current model unapplicable [9]. TFA and other strong ion-pairing agents potentially offer better chromatographic performance but at the cost of MS sensitivity. Nonetheless, we believe that the use of formic acid offers here a practical compromise with good chromatography and good MS sensitivity.

The models developed in the current study are particularly useful in case short peptides are detected that share similar MS and MS/MS spectra yet have different retention times. Our results clearly indicate the usefulness of predicted retention times and elution orders in assigning peptide identities.

## Conclusions

In this study, we accurately predicted the retention time of short peptides during RPLC-MS analysis, including peptide-sequence dependent effects. Sets of short peptides were prepared by swapped-sequence synthesis and protein digestion. The sample sets included many homologous tri- and tetrapeptide structures. We showed that next to the amino acid composition of a peptide, sequence dependent information must be included for accurate prediction of retention times. Amino acid index derived sequence-specific peptide (ASP) descriptors were developed to assess which information is essential. Using a step-wise variable selection procedure, 514 different sets of ASP-descriptors were evaluated and the most useful ones (BULH740102, QIAN880111) were added to the prediction model. The final prediction model had a goodness-of-fit (*Q*^2^) of 0.94; moreover, for 93% of the short peptide homologous pairs, the elution order was correctly predicted. The retention times of short peptides with additional charged side groups were more difficult to predict. Improving the predictability of these peptides was not in scope of the current study which focusses on short, hydrophobic peptides as these are most relevant for the taste sensation of foods. The predicted retention times can be used for assigning peptide identities.

## Supplementary Information

Below is the link to the electronic supplementary material.

Supplementary file1 (DOCX 98 KB)

## Declarations

BH and HGJ are employed by Unilever, a multinational company active in the field of foods, and home and personal care products.
