# Supplementary material for: Improved LC–MS identification of short homologous peptides using sequence-specific retention time predictors
Source: Anal Bioanal Chem. 2023 Mar 31;415(14):2715–26. doi: 10.1007/s00216-023-04670-2 (PMC10185643; doi:10.1007/s00216-023-04670-2)
Supplement: Supplementary file 1 — Supplementary file1 (DOCX 98 KB) [file 216_2023_4670_MOESM1_ESM.docx]

**Table S1. All peptide sequences and their retention times**

| **Peptide** | **RT** | **Source** | [M+H]^+^ | [M+2H]^2+^ |
| --- | --- | --- | --- | --- |
| **YNTG** | **3,1** | **1** | 454,1932 | 227,6003 |
| **YSTG** | **3,29** | **1** | 427,1823 | 214,0948 |
| **YNKV** | **3,33** | **1** | 523,2875 | 262,1474 |
| **YQTG** | **3,38** | **1** | 468,2089 | 234,6081 |
| **QTV** | **3,48** | **1** | 347,1925 | 174,0999 |
| **YSKV** | **3,63** | **1** | 496,2766 | 248,6419 |
| **QPV** | **3,88** | **1** | 343,1976 | 172,1024 |
| **RNLR** | **4,05** | **1** | 558,3470 | 279,6772 |
| **YQKV** | **4,06** | **1** | 537,3031 | 269,1552 |
| **NPV** | **4,11** | **1** | 329,1819 | 165,0946 |
| **YNPG** | **4,52** | **1** | 450,1983 | 225,6028 |
| **NTI** | **4,6** | **1** | 347,1925 | 174,0999 |
| **YNKI** | **4,67** | **1** | 537,3031 | 269,1552 |
| **YKV** | **4,69** | **1** | 409,2445 | 205,1259 |
| **YSKI** | **4,76** | **1** | 510,2922 | 255,6497 |
| **YSPG** | **4,78** | **1** | 423,1874 | 212,0973 |
| **YQPG** | **4,83** | **1** | 464,2140 | 232,6106 |
| **YPG** | **5,01** | **1** | 336,1554 | 168,5813 |
| **WNG** | **5,04** | **1** | 376,1615 | 188,5844 |
| **YQKI** | **5,11** | **1** | 551,3188 | 276,1630 |
| **WSG** | **5,23** | **1** | 349,1506 | 175,0790 |
| **QTI** | **5,27** | **1** | 361,2082 | 181,1077 |
| **WTG** | **5,38** | **1** | 363,1663 | 182,0868 |
| **WQG** | **5,4** | **1** | 390,1772 | 195,5922 |
| **WNKV** | **5,48** | **1** | 546,3034 | 273,6554 |
| **WSKV** | **5,52** | **1** | 519,2925 | 260,1499 |
| **QPI** | **5,62** | **1** | 357,2132 | 179,1103 |
| **NPI** | **5,88** | **1** | 343,1976 | 172,1024 |
| **WQKV** | **5,92** | **1** | 560,3191 | 280,6632 |
| **YKI** | **5,99** | **1** | 423,2602 | 212,1337 |
| **RVYV** | **6,1** | **1** | 536,3191 | 268,6632 |
| **WNTG** | **6,36** | **1** | 477,2092 | 239,1082 |
| **YNTV** | **6,41** | **1** | 496,2402 | 248,6237 |
| **YSTV** | **6,5** | **1** | 469,2293 | 235,1183 |
| **WQTG** | **6,52** | **1** | 491,2249 | 246,1161 |
| **WSTG** | **6,62** | **1** | 450,1983 | 225,6028 |
| **YQTV** | **6,97** | **1** | 510,2558 | 255,6316 |
| **WNKI** | **6,98** | **1** | 560,3191 | 280,6632 |
| **WSKI** | **7,04** | **1** | 533,3082 | 267,1577 |
| **YNV** | **7,35** | **1** | 395,1925 | 198,0999 |
| **WQKI** | **7,48** | **1** | 574,3347 | 287,6710 |
| **WKV** | **7,5** | **1** | 432,2605 | 216,6339 |
| **YQV** | **7,55** | **1** | 409,2082 | 205,1077 |
| **YSV** | **7,57** | **1** | 368,1816 | 184,5944 |
| **WNPG** | **7,62** | **1** | 473,2143 | 237,1108 |
| **YTV** | **7,66** | **1** | 382,1973 | 191,6023 |
| **YQPV** | **7,67** | **1** | 506,2609 | 253,6341 |
| **YNPV** | **8,02** | **1** | 492,2453 | 246,6263 |
| **WQPG** | **8,07** | **1** | 487,2299 | 244,1186 |
| **WSPG** | **8,07** | **1** | 446,2034 | 223,6053 |
| **YSPV** | **8,16** | **1** | 465,2344 | 233,1208 |
| **QYVL** | **8,23** | **1** | 522,2922 | 261,6497 |
| **YNTI** | **8,36** | **1** | 510,2558 | 255,6316 |
| **YSTI** | **8,47** | **1** | 483,2449 | 242,1261 |
| **YPV** | **8,54** | **1** | 378,2023 | 189,6048 |
| **RFFS** | **8,57** | **1** | 556,2878 | 278,6475 |
| **YQTI** | **8,92** | **1** | 524,2715 | 262,6394 |
| **WPG** | **9,42** | **1** | 359,1714 | 180,0893 |
| **YNI** | **9,5** | **1** | 409,2082 | 205,1077 |
| **WKI** | **9,65** | **1** | 446,2762 | 223,6417 |
| **YQPI** | **9,65** | **1** | 520,2766 | 260,6419 |
| **YQI** | **9,72** | **1** | 423,2238 | 212,1155 |
| **YSI** | **9,76** | **1** | 382,1973 | 191,6023 |
| **YTI** | **9,91** | **1** | 396,2129 | 198,6101 |
| **YNPI** | **10,03** | **1** | 506,2609 | 253,6341 |
| **WNTV** | **10,05** | **1** | 519,2562 | 260,1317 |
| **YSPI** | **10,14** | **1** | 479,2500 | 240,1286 |
| **WSTV** | **10,27** | **1** | 492,2453 | 246,6263 |
| **YPI** | **10,6** | **1** | 392,2180 | 196,6126 |
| **WQTV** | **10,65** | **1** | 533,2718 | 267,1395 |
| **WNV** | **11,55** | **1** | 418,2085 | 209,6079 |
| **WQPV** | **11,7** | **1** | 529,2769 | 265,1421 |
| **WNI** | **11,86** | **1** | 432,2241 | 216,6157 |
| **WNPV** | **11,86** | **1** | 515,2612 | 258,1343 |
| **WQV** | **11,86** | **1** | 432,2241 | 216,6157 |
| **WSV** | **11,93** | **1** | 391,1976 | 196,1024 |
| **WSI** | **12,09** | **1** | 405,2132 | 203,1103 |
| **WTV** | **12,1** | **1** | 405,2132 | 203,1103 |
| **WNTI** | **12,22** | **1** | 533,2718 | 267,1395 |
| **WSPV** | **12,33** | **1** | 488,2503 | 244,6288 |
| **WSTI** | **12,46** | **1** | 506,2609 | 253,6341 |
| **WQTI** | **12,76** | **1** | 547,2875 | 274,1474 |
| **WPV** | **13,46** | **1** | 401,2183 | 201,1128 |
| **WQPI** | **13,79** | **1** | 543,2925 | 272,1499 |
| **WNPI** | **14** | **1** | 529,2769 | 265,1421 |
| **WQI** | **14,27** | **1** | 446,2398 | 223,6235 |
| **WSPI** | **14,32** | **1** | 502,2660 | 251,6366 |
| **WTI** | **14,58** | **1** | 419,2289 | 210,1181 |
| **WPI** | **15,61** | **1** | 415,2340 | 208,1206 |
| **YIKS** | **3,09** | **2** | 510,2922 | 255,6497 |
| **YGTN** | **3,1** | **2** | 454,1932 | 227,6003 |
| **YIKQ** | **3,48** | **2** | 551,3188 | 276,1630 |
| **WGKN** | **3,54** | **2** | 504,2565 | 252,6319 |
| **YGTS** | **3,58** | **2** | 427,1823 | 214,0948 |
| **WGKS** | **3,7** | **2** | 477,2456 | 239,1264 |
| **YGTQ** | **3,75** | **2** | 468,2089 | 234,6081 |
| **WGKQ** | **3,83** | **2** | 518,2721 | 259,6397 |
| **WVKN** | **4,18** | **2** | 546,3034 | 273,6554 |
| **WVKS** | **4,49** | **2** | 519,2925 | 260,1499 |
| **WVKQ** | **4,62** | **2** | 560,3191 | 280,6632 |
| **YVTN** | **4,99** | **2** | 496,2402 | 248,6237 |
| **YGPN** | **5,31** | **2** | 450,1983 | 225,6028 |
| **YVTS** | **5,31** | **2** | 469,2293 | 235,1183 |
| **YVTQ** | **5,34** | **2** | 510,2558 | 255,6316 |
| **WIKN** | **5,44** | **2** | 560,3191 | 280,6632 |
| **YGPS** | **5,51** | **2** | 423,1874 | 212,0973 |
| **WIKS** | **5,68** | **2** | 533,3082 | 267,1577 |
| **WIKQ** | **5,7** | **2** | 574,3347 | 287,6710 |
| **YGPQ** | **5,76** | **2** | 464,2140 | 232,6106 |
| **YVPN** | **5,88** | **2** | 492,2453 | 246,6263 |
| **YVPS** | **6,21** | **2** | 465,2344 | 233,1208 |
| **YVPQ** | **6,26** | **2** | 506,2609 | 253,6341 |
| **YITN** | **6,55** | **2** | 510,2558 | 255,6316 |
| **YITQ** | **6,76** | **2** | 524,2715 | 262,6394 |
| **YITS** | **6,84** | **2** | 483,2449 | 242,1261 |
| **WGTN** | **6,86** | **2** | 477,2092 | 239,1082 |
| **WGTQ** | **7,03** | **2** | 491,2249 | 246,1161 |
| **WGTS** | **7,06** | **2** | 450,1983 | 225,6028 |
| **YIPN** | **7,3** | **2** | 506,2609 | 253,6341 |
| **YIPQ** | **7,63** | **2** | 520,2766 | 260,6419 |
| **YIPS** | **7,7** | **2** | 479,2500 | 240,1286 |
| **WGPN** | **8,41** | **2** | 473,2143 | 237,1108 |
| **WVTN** | **8,53** | **2** | 519,2562 | 260,1317 |
| **WGPS** | **8,64** | **2** | 446,2034 | 223,6053 |
| **WVTQ** | **8,75** | **2** | 533,2718 | 267,1395 |
| **WGPQ** | **8,81** | **2** | 487,2299 | 244,1186 |
| **WVTS** | **8,89** | **2** | 492,2453 | 246,6263 |
| **WVPN** | **9,34** | **2** | 515,2612 | 258,1343 |
| **WVPQ** | **9,63** | **2** | 529,2769 | 265,1421 |
| **WVPS** | **9,71** | **2** | 488,2503 | 244,6288 |
| **WITN** | **10,66** | **2** | 533,2718 | 267,1395 |
| **WITQ** | **10,72** | **2** | 547,2875 | 274,1474 |
| **WIPN** | **10,97** | **2** | 529,2769 | 265,1421 |
| **WITS** | **11,04** | **2** | 506,2609 | 253,6341 |
| **WIPQ** | **11,21** | **2** | 543,2925 | 272,1499 |
| **WIPS** | **11,4** | **2** | 502,2660 | 251,6366 |
| **PRY** | **3,11** | **3** | 435,2350 | 218,1212 |
| **PTRY** | **3,15** | **3** | 536,2827 | 268,6450 |
| **FTRA** | **3,4** | **3** | 494,2721 | 247,6397 |
| **TRA** | **3,42** | **3** | 347,2037 | 174,1055 |
| **PDRY** | **3,54** | **3** | 550,2620 | 275,6346 |
| **PERY** | **3,65** | **3** | 564,2776 | 282,6425 |
| **FDRA** | **3,81** | **3** | 508,2514 | 254,6293 |
| **FERA** | **4** | **3** | 522,2671 | 261,6372 |
| **FRA** | **4,07** | **3** | 393,2245 | 197,1159 |
| **TIA** | **4,16** | **3** | 304,1867 | 152,5970 |
| **TGY** | **4,3** | **3** | 340,1503 | 170,5788 |
| **EIA** | **4,46** | **3** | 332,1816 | 166,5944 |
| **PIA** | **4,64** | **3** | 300,1918 | 150,5995 |
| **EGY** | **4,66** | **3** | 368,1452 | 184,5762 |
| **DGY** | **4,72** | **3** | 354,1296 | 177,5684 |
| **TRW** | **4,92** | **3** | 462,2459 | 231,6266 |
| **PGY** | **5,02** | **3** | 336,1554 | 168,5813 |
| **ERW** | **5,25** | **3** | 490,2408 | 245,6241 |
| **DIA** | **5,31** | **3** | 318,1659 | 159,5866 |
| **PTGY** | **5,36** | **3** | 437,2031 | 219,1052 |
| **PTRW** | **5,45** | **3** | 559,2987 | 280,1530 |
| **PTY** | **5,48** | **3** | 380,1816 | 190,5944 |
| **PRW** | **5,5** | **3** | 458,2510 | 229,6291 |
| **FTRY** | **5,57** | **3** | 586,2984 | 293,6528 |
| **FTGA** | **5,64** | **3** | 395,1925 | 198,0999 |
| **PEY** | **5,73** | **3** | 408,1765 | 204,5919 |
| **PDY** | **5,77** | **3** | 394,1609 | 197,5841 |
| **PERW** | **5,81** | **3** | 587,2936 | 294,1504 |
| **PDRW** | **5,88** | **3** | 573,2780 | 287,1426 |
| **FGA** | **5,99** | **3** | 294,1448 | 147,5760 |
| **FDGA** | **6,03** | **3** | 409,1718 | 205,0895 |
| **FERY** | **6,03** | **3** | 614,2933 | 307,6503 |
| **PEGY** | **6,03** | **3** | 465,1980 | 233,1026 |
| **PDGY** | **6,08** | **3** | 451,1823 | 226,0948 |
| **FDRY** | **6,16** | **3** | 600,2776 | 300,6425 |
| **FEGA** | **6,27** | **3** | 423,1874 | 212,0973 |
| **FTA** | **6,31** | **3** | 338,1710 | 169,5892 |
| **PTIA** | **6,41** | **3** | 401,2394 | 201,1234 |
| **FDA** | **6,61** | **3** | 352,1503 | 176,5788 |
| **FRY** | **6,66** | **3** | 485,2507 | 243,1290 |
| **PGA** | **6,67** | **3** | 244,1292 | 122,5682 |
| **PDIA** | **6,69** | **3** | 415,2187 | 208,1130 |
| **FEA** | **6,71** | **3** | 366,1659 | 183,5866 |
| **PEIA** | **6,75** | **3** | 429,2344 | 215,1208 |
| **TGW** | **8,01** | **3** | 363,1663 | 182,0868 |
| **PERA** | **8,02** | **3** | 472,2514 | 236,6293 |
| **TIY** | **8,11** | **3** | 396,2129 | 198,6101 |
| **FTRW** | **8,45** | **3** | 609,3143 | 305,1608 |
| **EGW** | **8,53** | **3** | 391,1612 | 196,0842 |
| **EIY** | **8,66** | **3** | 424,2078 | 212,6075 |
| **PGW** | **8,86** | **3** | 359,1714 | 180,0893 |
| **PIY** | **8,92** | **3** | 392,2180 | 196,6126 |
| **FERW** | **9,02** | **3** | 637,3093 | 319,1583 |
| **FIA** | **9,07** | **3** | 350,2074 | 175,6073 |
| **PTGW** | **9,17** | **3** | 460,2190 | 230,6132 |
| **FDRW** | **9,24** | **3** | 623,2936 | 312,1504 |
| **DGW** | **9,29** | **3** | 377,1455 | 189,0764 |
| **FTGY** | **9,35** | **3** | 487,2187 | 244,1130 |
| **PTW** | **9,39** | **3** | 403,1976 | 202,1024 |
| **PEGW** | **9,79** | **3** | 488,2140 | 244,6106 |
| **DIY** | **9,9** | **3** | 410,1922 | 205,5997 |
| **PEW** | **9,9** | **3** | 431,1925 | 216,0999 |
| **PDW** | **9,91** | **3** | 417,1768 | 209,0921 |
| **PDGW** | **9,93** | **3** | 474,1983 | 237,6028 |
| **FDGY** | **9,96** | **3** | 501,1980 | 251,1026 |
| **FEGY** | **10,02** | **3** | 515,2136 | 258,1105 |
| **FGY** | **10,05** | **3** | 386,1710 | 193,5892 |
| **PTIY** | **10,12** | **3** | 493,2657 | 247,1365 |
| **PDIY** | **10,36** | **3** | 507,2449 | 254,1261 |
| **PEIY** | **10,39** | **3** | 521,2606 | 261,1339 |
| **FTY** | **10,42** | **3** | 430,1973 | 215,6023 |
| **FRW** | **10,44** | **3** | 508,2667 | 254,6370 |
| **FEY** | **10,71** | **3** | 458,1922 | 229,5997 |
| **FDY** | **10,77** | **3** | 444,1765 | 222,5919 |
| **FTIA** | **12,24** | **3** | 451,2551 | 226,1312 |
| **FEIA** | **12,57** | **3** | 479,2500 | 240,1286 |
| **FDIA** | **12,6** | **3** | 465,2344 | 233,1208 |
| **TIW** | **12,99** | **3** | 419,2289 | 210,1181 |
| **FIY** | **13,07** | **3** | 442,2336 | 221,6205 |
| **PDRA** | **13,09** | **3** | 458,2358 | 229,6215 |
| **EIW** | **13,53** | **3** | 447,2238 | 224,1155 |
| **FTGW** | **13,72** | **3** | 510,2347 | 255,6210 |
| **PIW** | **13,98** | **3** | 415,2340 | 208,1206 |
| **FEGW** | **14,25** | **3** | 538,2296 | 269,6184 |
| **FDGW** | **14,26** | **3** | 524,2140 | 262,6106 |
| **PTIW** | **14,68** | **3** | 516,2816 | 258,6445 |
| **FGW** | **14,82** | **3** | 409,1870 | 205,0971 |
| **PEIW** | **14,86** | **3** | 544,2766 | 272,6419 |
| **PDIW** | **14,97** | **3** | 530,2609 | 265,6341 |
| **DIW** | **15,15** | **3** | 433,2081 | 217,1077 |
| **FTW** | **15,17** | **3** | 453,2132 | 227,1103 |
| **FTIY** | **15,26** | **3** | 543,2813 | 272,1443 |
| **FDIY** | **15,45** | **3** | 557,2606 | 279,1339 |
| **FDW** | **15,54** | **3** | 467,1925 | 234,0999 |
| **FEIY** | **15,54** | **3** | 571,2762 | 286,1418 |
| **FEW** | **15,54** | **3** | 481,2081 | 241,1077 |
| **FIW** | **18,05** | **3** | 465,2496 | 233,1284 |
| **FTIW** | **19,37** | **3** | 566,2973 | 283,6523 |
| **FEIW** | **19,54** | **3** | 594,2922 | 297,6497 |
| **FDIW** | **19,61** | **3** | 580,2766 | 290,6419 |
| **PYRT** | **3,15** | **4** | 536,2827 | 268,6450 |
| **FARD** | **3,43** | **4** | 508,2514 | 254,6293 |
| **FART** | **3,57** | **4** | 494,2721 | 247,6397 |
| **FARE** | **4** | **4** | 522,2671 | 261,6372 |
| **PYGD** | **4,84** | **4** | 451,1823 | 226,0948 |
| **PWRD** | **5,01** | **4** | 573,2780 | 287,1426 |
| **PWRE** | **5,25** | **4** | 587,2936 | 294,1504 |
| **PWRT** | **5,3** | **4** | 559,2987 | 280,1530 |
| **PYGT** | **5,36** | **4** | 437,2031 | 219,1052 |
| **FYRD** | **5,39** | **4** | 600,2776 | 300,6425 |
| **FAGD** | **5,49** | **4** | 409,1718 | 205,0895 |
| **FYRT** | **5,49** | **4** | 586,2984 | 293,6528 |
| **FYRE** | **5,6** | **4** | 614,2933 | 307,6503 |
| **PYGE** | **5,63** | **4** | 465,1980 | 233,1026 |
| **FAGT** | **5,93** | **4** | 395,1925 | 198,0999 |
| **PAIT** | **6,04** | **4** | 401,2394 | 201,1234 |
| **PAID** | **6,25** | **4** | 415,2187 | 208,1130 |
| **FAGE** | **6,27** | **4** | 423,1874 | 212,0973 |
| **PAIE** | **6,75** | **4** | 429,2344 | 215,1208 |
| **PARE** | **8,02** | **4** | 472,2514 | 236,6293 |
| **FWRD** | **8,16** | **4** | 623,2936 | 312,1504 |
| **PWGD** | **8,27** | **4** | 474,1983 | 237,6028 |
| **FWRE** | **8,3** | **4** | 637,3093 | 319,1583 |
| **FWRT** | **8,45** | **4** | 609,3143 | 305,1608 |
| **PWGT** | **8,77** | **4** | 460,2190 | 230,6132 |
| **PWGE** | **8,84** | **4** | 488,2140 | 244,6106 |
| **FYGD** | **8,93** | **4** | 501,1980 | 251,1026 |
| **PYIT** | **9,16** | **4** | 493,2657 | 247,1365 |
| **FYGT** | **9,35** | **4** | 487,2187 | 244,1130 |
| **FYGE** | **9,43** | **4** | 515,2136 | 258,1105 |
| **PYID** | **9,52** | **4** | 507,2449 | 254,1261 |
| **PYIE** | **9,52** | **4** | 521,2606 | 261,1339 |
| **FAIT** | **11,84** | **4** | 451,2551 | 226,1312 |
| **FAID** | **12,21** | **4** | 465,2344 | 233,1208 |
| **FAIE** | **12,28** | **4** | 479,2500 | 240,1286 |
| **PARD** | **13,09** | **4** | 458,2358 | 229,6215 |
| **FWGD** | **13,14** | **4** | 524,2140 | 262,6106 |
| **FWGT** | **13,24** | **4** | 510,2347 | 255,6210 |
| **FYIT** | **13,31** | **4** | 543,2813 | 272,1443 |
| **PWIT** | **13,36** | **4** | 516,2816 | 258,6445 |
| **FWGE** | **13,38** | **4** | 538,2296 | 269,6184 |
| **PWIE** | **13,46** | **4** | 544,2766 | 272,6419 |
| **PWID** | **13,54** | **4** | 530,2609 | 265,6341 |
| **FYID** | **13,86** | **4** | 557,2606 | 279,1339 |
| **FYIE** | **13,87** | **4** | 571,2762 | 286,1418 |
| **FWIT** | **17,4** | **4** | 566,2973 | 283,6523 |
| **FWIE** | **17,52** | **4** | 594,2922 | 297,6497 |
| **FWID** | **17,72** | **4** | 580,2766 | 290,6419 |
| **SDIS** | **3,02** | **enzyme** | 421,1929 | 211,1001 |
| **KLV** | **3,06** | **enzyme** | 359,2653 | 180,1363 |
| **DAMS** | **3,07** | **enzyme** | 423,1544 | 212,0808 |
| **KIM** | **3,07** | **enzyme** | 391,2373 | 196,1223 |
| **IAA** | **3,08** | **enzyme** | 274,1761 | 137,5917 |
| **TPTTE** | **3,09** | **enzyme** | 548,2562 | 274,6318 |
| **PWAK** | **3,1** | **enzyme** | 501,2820 | 251,1446 |
| **EHFG** | **3,13** | **enzyme** | 489,2092 | 245,1082 |
| **KMY** | **3,14** | **enzyme** | 441,2166 | 221,1119 |
| **WKQ** | **3,14** | **enzyme** | 461,2507 | 231,1290 |
| **YGD** | **3,14** | **enzyme** | 354,1296 | 177,5684 |
| **TIAS** | **3,15** | **enzyme** | 391,2187 | 196,1130 |
| **TPEA** | **3,16** | **enzyme** | 417,1980 | 209,1026 |
| **SAYS** | **3,18** | **enzyme** | 427,1823 | 214,0948 |
| **DDSPD** | **3,22** | **enzyme** | 548,1834 | 274,5954 |
| **FRD** | **3,25** | **enzyme** | 437,2143 | 219,1108 |
| **YVN** | **3,3** | **enzyme** | 395,1925 | 198,0999 |
| **SLDG** | **3,37** | **enzyme** | 391,1823 | 196,0948 |
| **HIR** | **3,39** | **enzyme** | 425,2619 | 213,1346 |
| **DIS** | **3,44** | **enzyme** | 334,1609 | 167,5841 |
| **SLA** | **3,44** | **enzyme** | 290,1710 | 145,5892 |
| **GPKL** | **3,45** | **enzyme** | 414,2711 | 207,6392 |
| **SGYD** | **3,49** | **enzyme** | 441,1616 | 221,0844 |
| **AID** | **3,52** | **enzyme** | 318,1659 | 159,5866 |
| **TFRRP** | **3,54** | **enzyme** | 676,3889 | 338,6981 |
| **HPEY** | **3,61** | **enzyme** | 545,2354 | 273,1214 |
| **YDTQ** | **3,66** | **enzyme** | 526,2144 | 263,6108 |
| **LSP** | **3,71** | **enzyme** | 316,1867 | 158,5970 |
| **FRE** | **3,72** | **enzyme** | 451,2299 | 226,1186 |
| **TPVS** | **3,76** | **enzyme** | 403,2187 | 202,1130 |
| **AIT** | **3,83** | **enzyme** | 304,1867 | 152,5970 |
| **EAVE** | **3,83** | **enzyme** | 447,2085 | 224,1079 |
| **YGT** | **3,85** | **enzyme** | 340,1503 | 170,5788 |
| **AFDEK** | **3,86** | **enzyme** | 609,2878 | 305,1476 |
| **YVS** | **3,86** | **enzyme** | 368,1816 | 184,5944 |
| **YGE** | **3,99** | **enzyme** | 368,1452 | 184,5762 |
| **FRT** | **4,03** | **enzyme** | 423,2350 | 212,1212 |
| **EYE** | **4,05** | **enzyme** | 440,1663 | 220,5868 |
| **TVPQ** | **4,05** | **enzyme** | 444,2453 | 222,6263 |
| **YVQ** | **4,06** | **enzyme** | 409,2082 | 205,1077 |
| **QEHFG** | **4,07** | **enzyme** | 617,2678 | 309,1375 |
| **AIE** | **4,12** | **enzyme** | 332,1816 | 166,5944 |
| **PID** | **4,12** | **enzyme** | 344,1816 | 172,5944 |
| **PYD** | **4,12** | **enzyme** | 394,1609 | 197,5841 |
| **AFVK** | **4,14** | **enzyme** | 464,2867 | 232,6470 |
| **KKII** | **4,15** | **enzyme** | 501,3759 | 251,1916 |
| **EELK** | **4,16** | **enzyme** | 518,2820 | 259,6447 |
| **WRD** | **4,16** | **enzyme** | 476,2252 | 238,6162 |
| **SPV** | **4,17** | **enzyme** | 302,1710 | 151,5892 |
| **SAL** | **4,2** | **enzyme** | 290,1710 | 145,5892 |
| **VAP** | **4,2** | **enzyme** | 286,1761 | 143,5917 |
| **VLSN** | **4,27** | **enzyme** | 432,2453 | 216,6263 |
| **KLP** | **4,28** | **enzyme** | 357,2496 | 179,1284 |
| **ILDK** | **4,31** | **enzyme** | 488,3079 | 244,6576 |
| **HLVD** | **4,32** | **enzyme** | 483,2562 | 242,1317 |
| **ELT** | **4,33** | **enzyme** | 362,1922 | 181,5997 |
| **DEFK** | **4,35** | **enzyme** | 538,2507 | 269,6290 |
| **TLE** | **4,35** | **enzyme** | 362,1922 | 181,5997 |
| **AVFK** | **4,38** | **enzyme** | 464,2867 | 232,6470 |
| **KYLG** | **4,41** | **enzyme** | 480,2816 | 240,6445 |
| **HGFLK** | **4,45** | **enzyme** | 601,3456 | 301,1765 |
| **QKWE** | **4,46** | **enzyme** | 590,2933 | 295,6503 |
| **TEYG** | **4,46** | **enzyme** | 469,1929 | 235,1001 |
| **PIT** | **4,47** | **enzyme** | 330,2023 | 165,6048 |
| **AVVK** | **4,48** | **enzyme** | 416,2867 | 208,6470 |
| **PYE** | **4,49** | **enzyme** | 408,1765 | 204,5919 |
| **KCEVF** | **4,52** | **enzyme** | 682,3229 | 341,6651 |
| **ILNR** | **4,54** | **enzyme** | 515,3300 | 258,1686 |
| **WRE** | **4,55** | **enzyme** | 490,2408 | 245,6241 |
| **CLVR** | **4,57** | **enzyme** | 547,3021 | 274,1547 |
| **KIIA** | **4,59** | **enzyme** | 444,3180 | 222,6627 |
| **PYT** | **4,6** | **enzyme** | 380,1816 | 190,5944 |
| **DMAD** | **4,61** | **enzyme** | 451,1493 | 226,0783 |
| **VAVVK** | **4,61** | **enzyme** | 515,3551 | 258,1812 |
| **VKKIL** | **4,64** | **enzyme** | 600,4443 | 300,7258 |
| **QSL** | **4,65** | **enzyme** | 347,1925 | 174,0999 |
| **LLR** | **4,66** | **enzyme** | 401,2871 | 201,1472 |
| **PIE** | **4,66** | **enzyme** | 358,1972 | 179,6023 |
| **IPN** | **4,7** | **enzyme** | 343,1976 | 172,1024 |
| **VGP** | **4,7** | **enzyme** | 272,1605 | 136,5839 |
| **WRT** | **4,72** | **enzyme** | 462,2459 | 231,6266 |
| **LDAQS** | **4,75** | **enzyme** | 533,2566 | 267,1319 |
| **YPN** | **4,76** | **enzyme** | 393,1769 | 197,0921 |
| **WTN** | **4,81** | **enzyme** | 420,1878 | 210,5975 |
| **GVFK** | **4,83** | **enzyme** | 450,2711 | 225,6392 |
| **EEYVK** | **4,84** | **enzyme** | 667,3297 | 334,1685 |
| **IPEN** | **4,9** | **enzyme** | 472,2402 | 236,6237 |
| **PPK** | **4,91** | **enzyme** | 341,2183 | 171,1128 |
| **IPS** | **4,94** | **enzyme** | 316,1867 | 158,5970 |
| **IPQ** | **4,95** | **enzyme** | 357,2132 | 179,1103 |
| **QFL** | **4,98** | **enzyme** | 407,2289 | 204,1181 |
| **NLIK** | **5,03** | **enzyme** | 487,3238 | 244,1656 |
| **YPS** | **5,04** | **enzyme** | 366,1660 | 183,5866 |
| **AVVT** | **5,05** | **enzyme** | 389,2394 | 195,1234 |
| **DLPK** | **5,05** | **enzyme** | 472,2766 | 236,6419 |
| **YIN** | **5,08** | **enzyme** | 409,2082 | 205,1077 |
| **KDLK** | **5,17** | **enzyme** | 503,3188 | 252,1630 |
| **YPQ** | **5,17** | **enzyme** | 407,1925 | 204,0999 |
| **AIAA** | **5,19** | **enzyme** | 345,2132 | 173,1103 |
| **KII** | **5,19** | **enzyme** | 373,2809 | 187,1441 |
| **KYI** | **5,21** | **enzyme** | 423,2602 | 212,1337 |
| **KYL** | **5,22** | **enzyme** | 423,2602 | 212,1337 |
| **RYY** | **5,22** | **enzyme** | 501,2456 | 251,1264 |
| **QLL** | **5,24** | **enzyme** | 373,2445 | 187,1259 |
| **KIL** | **5,25** | **enzyme** | 373,2809 | 187,1441 |
| **TLED** | **5,25** | **enzyme** | 477,2191 | 239,1132 |
| **YVT** | **5,27** | **enzyme** | 382,1973 | 191,6023 |
| **GDLE** | **5,29** | **enzyme** | 433,1929 | 217,1001 |
| **WEN** | **5,29** | **enzyme** | 448,1827 | 224,5950 |
| **FGE** | **5,32** | **enzyme** | 352,1503 | 176,5788 |
| **FGD** | **5,33** | **enzyme** | 338,1346 | 169,5710 |
| **IRNL** | **5,33** | **enzyme** | 515,3300 | 258,1686 |
| **QYL** | **5,35** | **enzyme** | 423,2238 | 212,1155 |
| **ENKVL** | **5,38** | **enzyme** | 602,3508 | 301,6790 |
| **YIS** | **5,39** | **enzyme** | 382,1973 | 191,6023 |
| **YYQ** | **5,39** | **enzyme** | 473,2031 | 237,1052 |
| **PIQ** | **5,4** | **enzyme** | 357,2132 | 179,1103 |
| **WTS** | **5,43** | **enzyme** | 393,1769 | 197,0921 |
| **VAV** | **5,44** | **enzyme** | 288,1918 | 144,5995 |
| **WGN** | **5,45** | **enzyme** | 376,1615 | 188,5844 |
| **ESPPE** | **5,46** | **enzyme** | 558,2406 | 279,6239 |
| **WTQ** | **5,5** | **enzyme** | 434,2034 | 217,6053 |
| **YIQ** | **5,51** | **enzyme** | 423,2238 | 212,1155 |
| **KILD** | **5,53** | **enzyme** | 488,3079 | 244,6576 |
| **TVM** | **5,6** | **enzyme** | 350,1744 | 175,5908 |
| **EVFR** | **5,7** | **enzyme** | 550,2984 | 275,6528 |
| **WGS** | **5,71** | **enzyme** | 349,1506 | 175,0790 |
| **EQL** | **5,72** | **enzyme** | 389,2031 | 195,1052 |
| **VKF** | **5,75** | **enzyme** | 393,2496 | 197,1284 |
| **FGT** | **5,77** | **enzyme** | 324,1554 | 162,5813 |
| **LIN** | **5,77** | **enzyme** | 359,2289 | 180,1181 |
| **YAPE** | **5,8** | **enzyme** | 479,2136 | 240,1105 |
| **KEGYY** | **5,82** | **enzyme** | 659,3035 | 330,1554 |
| **LVNE** | **5,82** | **enzyme** | 474,2558 | 237,6316 |
| **VLA** | **5,83** | **enzyme** | 302,2074 | 151,6073 |
| **LINN** | **5,84** | **enzyme** | 473,2718 | 237,1395 |
| **FAE** | **5,87** | **enzyme** | 366,1659 | 183,5866 |
| **MDAKM** | **5,87** | **enzyme** | 595,2578 | 298,1326 |
| **ELLK** | **5,88** | **enzyme** | 502,3235 | 251,6654 |
| **FAD** | **5,89** | **enzyme** | 352,1503 | 176,5788 |
| **PLE** | **5,89** | **enzyme** | 358,1972 | 179,6023 |
| **VQVT** | **5,89** | **enzyme** | 446,2609 | 223,6341 |
| **AKYI** | **5,9** | **enzyme** | 494,2973 | 247,6523 |
| **EQLT** | **5,9** | **enzyme** | 490,2507 | 245,6290 |
| **MKGLD** | **5,9** | **enzyme** | 563,2857 | 282,1465 |
| **LPMH** | **5,95** | **enzyme** | 497,2541 | 249,1307 |
| **SPI** | **5,97** | **enzyme** | 316,1867 | 158,5970 |
| **TDTL** | **5,98** | **enzyme** | 449,2242 | 225,1157 |
| **FPKAE** | **6** | **enzyme** | 591,3137 | 296,1605 |
| **LINNQ** | **6,01** | **enzyme** | 601,3304 | 301,1688 |
| **WGQ** | **6,03** | **enzyme** | 390,1772 | 195,5922 |
| **FAT** | **6,05** | **enzyme** | 338,1710 | 169,5892 |
| **LLPK** | **6,05** | **enzyme** | 470,3337 | 235,6705 |
| **ELTE** | **6,08** | **enzyme** | 491,2348 | 246,1210 |
| **AIPEN** | **6,11** | **enzyme** | 543,2773 | 272,1423 |
| **IAVVK** | **6,13** | **enzyme** | 529,3708 | 265,1890 |
| **NNQF** | **6,13** | **enzyme** | 522,2307 | 261,6190 |
| **KWP** | **6,2** | **enzyme** | 430,2449 | 215,6261 |
| **KVLV** | **6,21** | **enzyme** | 458,3337 | 229,6705 |
| **LLQ** | **6,22** | **enzyme** | 373,2445 | 187,1259 |
| **KFF** | **6,23** | **enzyme** | 441,2496 | 221,1284 |
| **QVLS** | **6,27** | **enzyme** | 446,2609 | 223,6341 |
| **YVTA** | **6,27** | **enzyme** | 453,2344 | 227,1208 |
| **LWR** | **6,28** | **enzyme** | 474,2823 | 237,6448 |
| **KYLGE** | **6,29** | **enzyme** | 609,3242 | 305,1658 |
| **EALE** | **6,3** | **enzyme** | 461,2242 | 231,1157 |
| **IRAI** | **6,3** | **enzyme** | 472,3242 | 236,6657 |
| **QLR** | **6,31** | **enzyme** | 416,2616 | 208,6344 |
| **IRAL** | **6,32** | **enzyme** | 472,3242 | 236,6657 |
| **KASYL** | **6,32** | **enzyme** | 581,3293 | 291,1683 |
| **AIRNL** | **6,37** | **enzyme** | 586,3671 | 293,6872 |
| **IRAIA** | **6,37** | **enzyme** | 543,3613 | 272,1843 |
| **KLFT** | **6,39** | **enzyme** | 508,3129 | 254,6601 |
| **YID** | **6,4** | **enzyme** | 410,1922 | 205,5997 |
| **LIVR** | **6,43** | **enzyme** | 500,3555 | 250,6814 |
| **LVTD** | **6,48** | **enzyme** | 447,2449 | 224,1261 |
| **KIPA** | **6,5** | **enzyme** | 428,2867 | 214,6470 |
| **TVQVT** | **6,58** | **enzyme** | 547,3086 | 274,1579 |
| **AIVQ** | **6,63** | **enzyme** | 430,2660 | 215,6366 |
| **APNNL** | **6,67** | **enzyme** | 528,2776 | 264,6425 |
| **YIT** | **6,68** | **enzyme** | 396,2129 | 198,6101 |
| **DAKMY** | **6,69** | **enzyme** | 627,2807 | 314,1440 |
| **YIE** | **6,72** | **enzyme** | 424,2078 | 212,6075 |
| **DLTD** | **6,78** | **enzyme** | 463,2035 | 232,1054 |
| **TPEVD** | **6,78** | **enzyme** | 560,2562 | 280,6317 |
| **IIA** | **6,8** | **enzyme** | 316,2231 | 158,6152 |
| **LLA** | **6,8** | **enzyme** | 316,2231 | 158,6152 |
| **WGD** | **6,82** | **enzyme** | 377,1455 | 189,0764 |
| **SLVN** | **6,88** | **enzyme** | 432,2453 | 216,6263 |
| **VGIN** | **6,88** | **enzyme** | 402,2347 | 201,6210 |
| **WVN** | **6,89** | **enzyme** | 418,2085 | 209,6079 |
| **KKFW** | **6,91** | **enzyme** | 608,3555 | 304,6814 |
| **VFD** | **6,96** | **enzyme** | 380,1816 | 190,5944 |
| **ALPMH** | **7,01** | **enzyme** | 568,2912 | 284,6492 |
| **VLDT** | **7,01** | **enzyme** | 447,2449 | 224,1261 |
| **SAPL** | **7,02** | **enzyme** | 387,2238 | 194,1155 |
| **EKYL** | **7,03** | **enzyme** | 552,3028 | 276,6550 |
| **DPWAK** | **7,06** | **enzyme** | 616,3089 | 308,6581 |
| **SAGWN** | **7,1** | **enzyme** | 534,2307 | 267,6190 |
| **LPDT** | **7,14** | **enzyme** | 445,2293 | 223,1183 |
| **WGT** | **7,19** | **enzyme** | 363,1663 | 182,0868 |
| **LLYNK** | **7,22** | **enzyme** | 650,3872 | 325,6972 |
| **QAIVQ** | **7,25** | **enzyme** | 558,3246 | 279,6659 |
| **GFLK** | **7,26** | **enzyme** | 464,2867 | 232,6470 |
| **FNPT** | **7,31** | **enzyme** | 478,2296 | 239,6184 |
| **YVLSR** | **7,31** | **enzyme** | 637,3668 | 319,1870 |
| **WGE** | **7,33** | **enzyme** | 391,1612 | 196,0842 |
| **WVS** | **7,33** | **enzyme** | 391,1976 | 196,1024 |
| **KPLL** | **7,35** | **enzyme** | 470,3337 | 235,6705 |
| **WVQ** | **7,36** | **enzyme** | 432,2241 | 216,6157 |
| **KGYG** | **7,41** | **enzyme** | 424,2190 | 212,6132 |
| **YAVA** | **7,44** | **enzyme** | 423,2238 | 212,1155 |
| **KFL** | **7,48** | **enzyme** | 407,2653 | 204,1363 |
| **WLAH** | **7,52** | **enzyme** | 526,2772 | 263,6423 |
| **VNELT** | **7,53** | **enzyme** | 575,3035 | 288,1554 |
| **LLNQ** | **7,55** | **enzyme** | 487,2875 | 244,1474 |
| **FNPTQ** | **7,58** | **enzyme** | 606,2882 | 303,6477 |
| **LIA** | **7,59** | **enzyme** | 316,2231 | 158,6152 |
| **LPDTE** | **7,6** | **enzyme** | 574,2719 | 287,6396 |
| **DDLTD** | **7,61** | **enzyme** | 578,2304 | 289,6188 |
| **ELPT** | **7,61** | **enzyme** | 459,2449 | 230,1261 |
| **SPAQI** | **7,64** | **enzyme** | 515,2824 | 258,1448 |
| **IIAE** | **7,66** | **enzyme** | 445,2657 | 223,1365 |
| **PWD** | **7,66** | **enzyme** | 417,1768 | 209,0921 |
| **TAGWN** | **7,66** | **enzyme** | 548,2463 | 274,6268 |
| **EVIE** | **7,67** | **enzyme** | 489,2555 | 245,1314 |
| **KALPM** | **7,71** | **enzyme** | 559,3272 | 280,1672 |
| **PWT** | **7,72** | **enzyme** | 403,1976 | 202,1024 |
| **VAFVK** | **7,75** | **enzyme** | 563,3551 | 282,1812 |
| **KFLD** | **7,76** | **enzyme** | 522,2922 | 261,6497 |
| **PWE** | **7,78** | **enzyme** | 431,1925 | 216,0999 |
| **DDDLT** | **7,79** | **enzyme** | 578,2304 | 289,6188 |
| **DDDL** | **7,8** | **enzyme** | 477,1827 | 239,0950 |
| **KDLL** | **7,9** | **enzyme** | 488,3079 | 244,6576 |
| **SVL** | **7,9** | **enzyme** | 318,2023 | 159,6048 |
| **EGYYG** | **7,95** | **enzyme** | 588,2300 | 294,6186 |
| **HLFG** | **7,98** | **enzyme** | 473,2507 | 237,1290 |
| **VAL** | **7,98** | **enzyme** | 302,2074 | 151,6073 |
| **KIW** | **8,05** | **enzyme** | 446,2762 | 223,6417 |
| **ALVE** | **8,06** | **enzyme** | 431,2500 | 216,1286 |
| **LLD** | **8,14** | **enzyme** | 360,2129 | 180,6101 |
| **KMYLG** | **8,15** | **enzyme** | 611,3221 | 306,1647 |
| **KLF** | **8,16** | **enzyme** | 407,2653 | 204,1363 |
| **VYVE** | **8,17** | **enzyme** | 509,2606 | 255,1339 |
| **EKKFW** | **8,21** | **enzyme** | 737,3981 | 369,2027 |
| **FYD** | **8,26** | **enzyme** | 444,1765 | 222,5919 |
| **HLF** | **8,3** | **enzyme** | 416,2292 | 208,6182 |
| **SLV** | **8,3** | **enzyme** | 318,2023 | 159,6048 |
| **KDYEL** | **8,33** | **enzyme** | 667,3297 | 334,1685 |
| **LLRL** | **8,37** | **enzyme** | 514,3711 | 257,6892 |
| **VATL** | **8,4** | **enzyme** | 403,2551 | 202,1312 |
| **EVI** | **8,41** | **enzyme** | 360,2129 | 180,6101 |
| **NKIW** | **8,42** | **enzyme** | 560,3191 | 280,6632 |
| **TLPDT** | **8,42** | **enzyme** | 546,2770 | 273,6421 |
| **VTLD** | **8,42** | **enzyme** | 447,2449 | 224,1261 |
| **QILQ** | **8,44** | **enzyme** | 501,3031 | 251,1552 |
| **FYE** | **8,49** | **enzyme** | 458,1922 | 229,5997 |
| **HPYF** | **8,54** | **enzyme** | 563,2612 | 282,1343 |
| **FYT** | **8,63** | **enzyme** | 430,1973 | 215,6023 |
| **LVVS** | **8,63** | **enzyme** | 417,2707 | 209,1390 |
| **HGFL** | **8,75** | **enzyme** | 473,2507 | 237,1290 |
| **LGYE** | **8,75** | **enzyme** | 481,2293 | 241,1183 |
| **YFA** | **8,79** | **enzyme** | 400,1867 | 200,5970 |
| **KMYL** | **8,82** | **enzyme** | 554,3007 | 277,6540 |
| **FID** | **8,89** | **enzyme** | 394,1972 | 197,6023 |
| **HSTIF** | **8,89** | **enzyme** | 604,3089 | 302,6581 |
| **EGYY** | **8,91** | **enzyme** | 531,2085 | 266,1079 |
| **AGLV** | **8,94** | **enzyme** | 359,2289 | 180,1181 |
| **VAVV** | **8,94** | **enzyme** | 387,2602 | 194,1337 |
| **FIE** | **9,04** | **enzyme** | 408,2129 | 204,6101 |
| **IPIQ** | **9,04** | **enzyme** | 470,2973 | 235,6523 |
| **WPN** | **9,09** | **enzyme** | 416,1928 | 208,6001 |
| **YPSYG** | **9,13** | **enzyme** | 586,2507 | 293,6290 |
| **WIN** | **9,15** | **enzyme** | 432,2241 | 216,6157 |
| **WPS** | **9,15** | **enzyme** | 389,1819 | 195,0946 |
| **WPQ** | **9,19** | **enzyme** | 430,2085 | 215,6079 |
| **FIT** | **9,23** | **enzyme** | 380,2180 | 190,6126 |
| **CLV** | **9,27** | **enzyme** | 391,2010 | 196,1041 |
| **TELPT** | **9,28** | **enzyme** | 560,2926 | 280,6499 |
| **ELLN** | **9,32** | **enzyme** | 488,2715 | 244,6394 |
| **YSLA** | **9,38** | **enzyme** | 453,2344 | 227,1208 |
| **MSLD** | **9,45** | **enzyme** | 465,2014 | 233,1043 |
| **WIQ** | **9,48** | **enzyme** | 446,2398 | 223,6235 |
| **HTLF** | **9,49** | **enzyme** | 517,2769 | 259,1421 |
| **EILLQ** | **9,53** | **enzyme** | 615,3712 | 308,1892 |
| **VGF** | **9,57** | **enzyme** | 322,1761 | 161,5917 |
| **WIS** | **9,57** | **enzyme** | 405,2132 | 203,1103 |
| **ELLNQ** | **9,62** | **enzyme** | 616,3301 | 308,6687 |
| **EFVE** | **9,66** | **enzyme** | 523,2398 | 262,1236 |
| **VAF** | **9,72** | **enzyme** | 336,1918 | 168,5995 |
| **EFYG** | **9,79** | **enzyme** | 515,2136 | 258,1105 |
| **ERFFS** | **9,81** | **enzyme** | 685,3304 | 343,1688 |
| **IPIG** | **9,83** | **enzyme** | 399,2602 | 200,1337 |
| **SVLPS** | **9,86** | **enzyme** | 502,2871 | 251,6472 |
| **KDAFL** | **9,87** | **enzyme** | 593,3293 | 297,1683 |
| **AVAVV** | **9,94** | **enzyme** | 458,2973 | 229,6523 |
| **TVF** | **9,94** | **enzyme** | 366,2023 | 183,6048 |
| **WSVN** | **10,01** | **enzyme** | 505,2405 | 253,1239 |
| **LNYYQ** | **10,12** | **enzyme** | 700,3301 | 350,6687 |
| **ASYL** | **10,15** | **enzyme** | 453,2344 | 227,1208 |
| **LIVTQ** | **10,18** | **enzyme** | 573,3606 | 287,1840 |
| **YEYVT** | **10,27** | **enzyme** | 674,3032 | 337,6552 |
| **IPAV** | **10,36** | **enzyme** | 399,2602 | 200,1337 |
| **NQYFG** | **10,39** | **enzyme** | 628,2725 | 314,6399 |
| **AEFVE** | **10,41** | **enzyme** | 594,2769 | 297,6421 |
| **LLFK** | **10,42** | **enzyme** | 520,3493 | 260,6783 |
| **SFM** | **10,42** | **enzyme** | 384,1588 | 192,5830 |
| **LLP** | **10,48** | **enzyme** | 342,2387 | 171,6230 |
| **AEFYG** | **10,5** | **enzyme** | 586,2507 | 293,6290 |
| **DDIM** | **10,5** | **enzyme** | 493,1963 | 247,1018 |
| **KFW** | **10,54** | **enzyme** | 480,2605 | 240,6339 |
| **LIVT** | **10,54** | **enzyme** | 445,3020 | 223,1547 |
| **ENFV** | **10,65** | **enzyme** | 508,2402 | 254,6237 |
| **IAFS** | **10,69** | **enzyme** | 437,2394 | 219,1234 |
| **EIL** | **10,81** | **enzyme** | 374,2285 | 187,6179 |
| **TVLE** | **10,81** | **enzyme** | 461,2606 | 231,1339 |
| **WID** | **10,81** | **enzyme** | 433,2081 | 217,1077 |
| **IDAL** | **10,83** | **enzyme** | 431,2500 | 216,1286 |
| **LLFR** | **10,86** | **enzyme** | 548,3555 | 274,6814 |
| **LLYN** | **10,87** | **enzyme** | 522,2922 | 261,6497 |
| **SSLLE** | **10,89** | **enzyme** | 548,2926 | 274,6499 |
| **WIE** | **10,92** | **enzyme** | 447,2238 | 224,1155 |
| **SLL** | **10,96** | **enzyme** | 332,2180 | 166,6126 |
| **AEFV** | **10,98** | **enzyme** | 465,2344 | 233,1208 |
| **SLLE** | **10,98** | **enzyme** | 461,2606 | 231,1339 |
| **KWF** | **11** | **enzyme** | 480,2605 | 240,6339 |
| **TWYS** | **11,02** | **enzyme** | 556,2402 | 278,6237 |
| **TIFEN** | **11,04** | **enzyme** | 623,3035 | 312,1554 |
| **TIL** | **11,11** | **enzyme** | 346,2336 | 173,6205 |
| **DKFL** | **11,16** | **enzyme** | 522,2922 | 261,6497 |
| **MYLG** | **11,17** | **enzyme** | 483,2272 | 242,1172 |
| **ERFF** | **11,19** | **enzyme** | 598,2984 | 299,6528 |
| **WIT** | **11,24** | **enzyme** | 419,2289 | 210,1181 |
| **SSLL** | **11,29** | **enzyme** | 419,2500 | 210,1286 |
| **LAL** | **11,32** | **enzyme** | 316,2231 | 158,6152 |
| **LAI** | **11,38** | **enzyme** | 316,2231 | 158,6152 |
| **LNYY** | **11,38** | **enzyme** | 572,2715 | 286,6394 |
| **NQYF** | **11,49** | **enzyme** | 571,2511 | 286,1292 |
| **VSVL** | **11,59** | **enzyme** | 417,2707 | 209,1390 |
| **QIL** | **11,66** | **enzyme** | 373,2445 | 187,1259 |
| **AEFY** | **11,69** | **enzyme** | 529,2293 | 265,1183 |
| **LDQW** | **11,75** | **enzyme** | 561,2667 | 281,1370 |
| **YVL** | **11,75** | **enzyme** | 394,2336 | 197,6205 |
| **PQTFY** | **11,79** | **enzyme** | 655,3086 | 328,1579 |
| **AGYF** | **11,81** | **enzyme** | 457,2081 | 229,1077 |
| **ELL** | **11,87** | **enzyme** | 374,2285 | 187,6179 |
| **LIV** | **11,98** | **enzyme** | 344,2544 | 172,6308 |
| **VPVLA** | **12** | **enzyme** | 498,3286 | 249,6679 |
| **LILN** | **12,01** | **enzyme** | 472,3129 | 236,6601 |
| **FYYA** | **12,05** | **enzyme** | 563,2500 | 282,1286 |
| **GDLL** | **12,13** | **enzyme** | 417,2344 | 209,1208 |
| **SLLEA** | **12,27** | **enzyme** | 532,2977 | 266,6525 |
| **LLY** | **12,31** | **enzyme** | 408,2493 | 204,6283 |
| **FWE** | **12,32** | **enzyme** | 481,2081 | 241,1077 |
| **LVL** | **12,35** | **enzyme** | 344,2544 | 172,6308 |
| **FWD** | **12,4** | **enzyme** | 467,1925 | 234,0999 |
| **FWT** | **12,48** | **enzyme** | 453,2132 | 227,1103 |
| **ALIVT** | **12,49** | **enzyme** | 516,3392 | 258,6732 |
| **NQFL** | **12,53** | **enzyme** | 521,2718 | 261,1395 |
| **SGLNL** | **12,61** | **enzyme** | 503,2824 | 252,1448 |
| **TLFG** | **12,63** | **enzyme** | 437,2394 | 219,1234 |
| **DVAF** | **12,75** | **enzyme** | 451,2187 | 226,1130 |
| **VPVL** | **12,87** | **enzyme** | 427,2915 | 214,1494 |
| **SFL** | **12,89** | **enzyme** | 366,2023 | 183,6048 |
| **GLLYN** | **12,91** | **enzyme** | 579,3137 | 290,1605 |
| **VLI** | **12,92** | **enzyme** | 344,2544 | 172,6308 |
| **DYEL** | **12,97** | **enzyme** | 539,2348 | 270,1210 |
| **EFFS** | **13,08** | **enzyme** | 529,2293 | 265,1183 |
| **TLF** | **13,21** | **enzyme** | 380,2180 | 190,6126 |
| **GDLEI** | **13,38** | **enzyme** | 546,2769 | 273,6421 |
| **NFFS** | **13,38** | **enzyme** | 514,2296 | 257,6184 |
| **NFFSG** | **13,44** | **enzyme** | 571,2511 | 286,1292 |
| **ALIV** | **13,53** | **enzyme** | 415,2915 | 208,1494 |
| **DERFF** | **13,56** | **enzyme** | 713,3253 | 357,1663 |
| **LLLA** | **13,56** | **enzyme** | 429,3071 | 215,1572 |
| **CPFD** | **13,62** | **enzyme** | 538,1966 | 269,6019 |
| **DLLFK** | **13,68** | **enzyme** | 635,3763 | 318,1918 |
| **CFL** | **13,89** | **enzyme** | 439,2010 | 220,1041 |
| **LWP** | **13,95** | **enzyme** | 415,2340 | 208,1206 |
| **DLLFR** | **14,11** | **enzyme** | 663,3824 | 332,1948 |
| **KDLLF** | **14,22** | **enzyme** | 635,3763 | 318,1918 |
| **LLL** | **14,37** | **enzyme** | 358,2700 | 179,6386 |
| **GLLY** | **14,49** | **enzyme** | 465,2707 | 233,1390 |
| **SDLTW** | **14,84** | **enzyme** | 621,2879 | 311,1476 |
| **INYW** | **14,93** | **enzyme** | 595,2875 | 298,1474 |
| **DLEI** | **14,95** | **enzyme** | 489,2555 | 245,1314 |
| **DAFL** | **15,15** | **enzyme** | 465,2344 | 233,1208 |
| **FPQL** | **15,15** | **enzyme** | 504,2816 | 252,6445 |
| **MGLL** | **15,22** | **enzyme** | 433,2479 | 217,1276 |
| **LPEW** | **15,32** | **enzyme** | 544,2766 | 272,6419 |
| **VLVL** | **15,55** | **enzyme** | 443,3228 | 222,1650 |
| **LIWE** | **15,85** | **enzyme** | 560,3079 | 280,6576 |
| **IGLL** | **15,99** | **enzyme** | 415,2915 | 208,1494 |
| **EILL** | **16** | **enzyme** | 487,3126 | 244,1599 |
| **LLQW** | **16,2** | **enzyme** | 559,3238 | 280,1656 |
| **FVYI** | **16,62** | **enzyme** | 541,3020 | 271,1547 |
| **LEIL** | **16,63** | **enzyme** | 487,3126 | 244,1599 |
| **WQVL** | **16,74** | **enzyme** | 545,3082 | 273,1577 |
| **YGLF** | **16,9** | **enzyme** | 499,2551 | 250,1312 |
| **LIW** | **17,09** | **enzyme** | 431,2653 | 216,1363 |
| **WELLN** | **17,11** | **enzyme** | 674,3508 | 337,6790 |
| **FQLFS** | **17,91** | **enzyme** | 641,3293 | 321,1683 |
| **WNIPM** | **18,28** | **enzyme** | 660,3174 | 330,6623 |
| **EDLIW** | **19,16** | **enzyme** | 675,3348 | 338,1710 |
| **LWF** | **19,6** | **enzyme** | 465,2496 | 233,1284 |
| **DLLF** | **19,88** | **enzyme** | 507,2813 | 254,1443 |
| **FLLF** | **20,08** | **enzyme** | 539,3228 | 270,1650 |
| **EFQLF** | **20,34** | **enzyme** | 683,3399 | 342,1736 |
| **LLLF** | **20,85** | **enzyme** | 505,3384 | 253,1729 |
| **LIWEL** | **21,65** | **enzyme** | 673,3919 | 337,1996 |
| **KAF** | **3,24** | **Tripep** | 365,2183 | 183,1128 |
| **AKF** | **3,5** | **Tripep** | 365,2183 | 183,1128 |
| **LKP** | **3,79** | **Tripep** | 357,2496 | 179,1284 |
| **AWR** | **4,1** | **Tripep** | 432,2354 | 216,6213 |
| **GWR** | **4,17** | **Tripep** | 418,2197 | 209,6135 |
| **VWR** | **4,73** | **Tripep** | 460,2667 | 230,6370 |
| **GGF** | **5,31** | **Tripep** | 280,1292 | 140,5682 |
| **LAP** | **6,01** | **Tripep** | 300,1918 | 150,5995 |
| **AGF** | **6,23** | **Tripep** | 294,1448 | 147,5760 |
| **GAF** | **6,23** | **Tripep** | 294,1448 | 147,5760 |
| **LGP** | **6,61** | **Tripep** | 286,1761 | 143,5917 |
| **ALP** | **7,15** | **Tripep** | 300,1918 | 150,5995 |
| **GLP** | **7,4** | **Tripep** | 286,1761 | 143,5917 |
| **LKF** | **7,74** | **Tripep** | 407,2653 | 204,1363 |
| **VLP** | **8,1** | **Tripep** | 328,2231 | 164,6152 |
| **AWP** | **10,5** | **Tripep** | 373,1870 | 187,0971 |
| **GWP** | **10,6** | **Tripep** | 359,1714 | 180,0893 |
| **VWP** | **11,62** | **Tripep** | 401,2183 | 201,1128 |
| **LGF** | **12,25** | **Tripep** | 336,1918 | 168,5995 |
| **LAF** | **12,8** | **Tripep** | 350,2074 | 175,6073 |
| **ALF** | **13,4** | **Tripep** | 350,2074 | 175,6073 |
| **GLF** | **13,47** | **Tripep** | 336,1918 | 168,5995 |
| **VLF** | **14,35** | **Tripep** | 378,2387 | 189,6230 |
| **GWF** | **16,17** | **Tripep** | 409,1870 | 205,0971 |
| **AWF** | **16,25** | **Tripep** | 423,2027 | 212,1050 |
| **LLF** | **16,93** | **Tripep** | 392,2544 | 196,6308 |
| **VWF** | **17,32** | **Tripep** | 451,2340 | 226,1206 |
|  |  |  |  |  |
|  |  |  |  |  |
|  |  |  |  |  |
|  |  |  |  |  |
|  |  |  |  |  |
|  |  |  |  |  |
|  |  |  |  |  |
|  |  |  |  |  |
|  |  |  |  |  |
|  |  |  |  |  |
|  |  |  |  |  |
|  |  |  |  |  |
|  |  |  |  |  |
|  |  |  |  |  |
|  |  |  |  |  |
|  |  |  |  |  |
|  |  |  |  |  |
|  |  |  |  |  |
|  |  |  |  |  |
|  |  |  |  |  |
|  |  |  |  |  |
|  |  |  |  |  |
|  |  |  |  |  |
|  |  |  |  |  |
|  |  |  |  |  |
|  |  |  |  |  |
|  |  |  |  |  |
|  |  |  |  |  |
|  |  |  |  |  |
|  |  |  |  |  |
|  |  |  |  |  |
|  |  |  |  |  |
|  |  |  |  |  |
|  |  |  |  |  |
|  |  |  |  |  |
|  |  |  |  |  |
|  |  |  |  |  |
|  |  |  |  |  |
|  |  |  |  |  |
|  |  |  |  |  |
|  |  |  |  |  |
|  |  |  |  |  |
|  |  |  |  |  |
|  |  |  |  |  |
|  |  |  |  |  |
|  |  |  |  |  |
